# Comparison of in vitro approaches for predicting the metabolism of the selective androgen receptor modulator RAD140

**DOI:** 10.1007/s00216-023-04835-z

**Published:** 2023-07-08

**Authors:** Felicitas Wagener, Nana Naumann, Valentin Göldner, Christian Görgens, Sven Guddat, Uwe Karst, Mario Thevis

**Affiliations:** 1 Center for Preventive Doping Research/Institute of Biochemistry, German Sport University Cologne, Am Sportpark Müngersdorf 6, 50933 Cologne, Germany; 2 Institute of Inorganic and Analytical Chemistry, University of Münster, Münster, Germany; 3 International Graduate School for Battery Chemistry, Characterization, Analysis, Recycling and Application (BACCARA), University of Münster, Münster, Germany; 4 European Monitoring Center for Emerging Doping Agents (EuMoCEDA), Cologne, Germany

**Keywords:** Organ on a chip, Metabolism, Doping, HepaRG, Electrochemistry, Sport

## Abstract

The identification of metabolites allows for the expansion of possible targets for anti-doping analysis. Especially for novel substances such as selective androgen receptor modulators (SARMs), information on metabolic fate is scarce. Novel approaches such as the organ on a chip technology may provide a metabolic profile that resembles human in vivo samples more closely than approaches that rely on human liver fractions only. In this study, the SARM RAD140 was metabolized by means of subcellular human liver fractions, human liver spheroids in an organ on a chip platform, and electrochemical (EC) conversion. The resulting metabolites were analyzed with LC-HRMS/MS and compared to a human doping control urine sample that yielded an adverse analytical finding for RAD140. A total of 16 metabolites were detected in urine, while 14, 13, and 7 metabolites were detected in samples obtained from the organ on a chip experiment, the subcellular liver fraction, and EC experiments, respectively. All tested techniques resulted in the detection of RAD140 metabolites. In the organ on a chip samples, the highest number of metabolites were detected. The subcellular liver fractions and organ on a chip techniques are deemed complementary to predict metabolites of RAD140, as both techniques produce distinct metabolites that are also found in an anonymized human in vivo urine sample.

**Graphical abstract:**

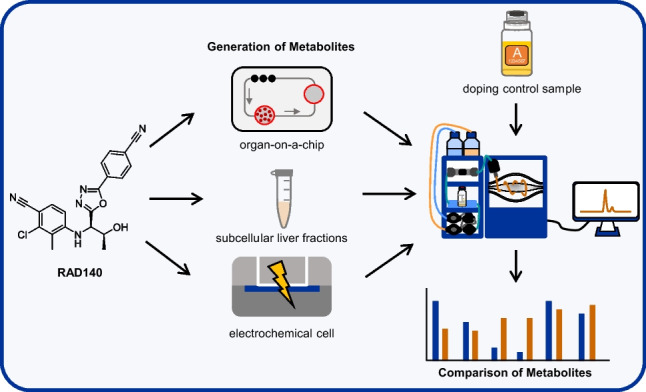

**Supplementary Information:**

The online version contains supplementary material available at 10.1007/s00216-023-04835-z.

## Introduction

**Fig. 1:**
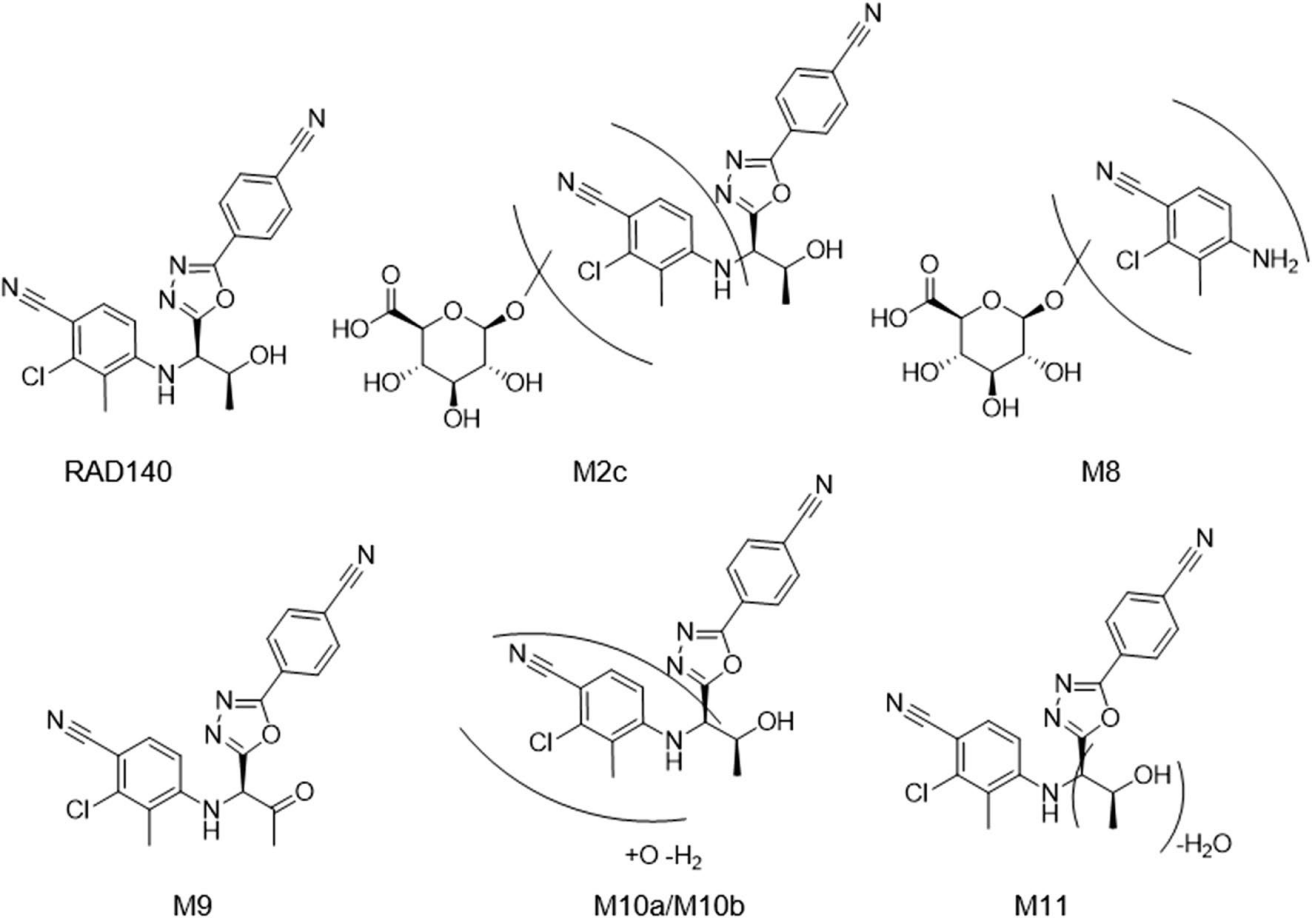
Structure of RAD140 and proposed structures of the newly described metabolites of RAD140, M2c, M8, M9, M10a, M10b, and M11. The HRMS/MS spectra can be found in Electronic supplementary material Figs. 22–27. The structures of the previously described metabolites can be found in Electronic supplementary material Fig. 28

RAD140 is a selective androgen receptor modulator (SARM) that is being investigated as a drug candidate for the treatment of breast cancer (Fig. 1) [1, 2]. Due to its potential performance-enhancing effect, research focusing on the detection of RAD140 for doping control purposes has been conducted in the past 10 years [3, 4]. Since 2016, a total of 24 adverse analytical findings (AAF) were reported for RAD140, which makes it the third most detected SARM after ostarine and LGD-4033 [5–10]. For doping control purposes, it is crucial to enable the detection of not only the prohibited substances themselves but also their metabolites, as many substances are mainly excreted in the metabolized form [11, 12]. There are multiple approaches to gain insight into the metabolic behavior of a substance, such as in vitro or in vivo techniques. As the metabolic fate may vary between species, human in vivo studies are the gold standard for human doping controls. But as human studies with unapproved drug candidates pose health risks to volunteers [13], alternative methods expanding the knowledge about the metabolic fate of doping agents are an important field of research. Different techniques of drug transformation show unique advantages and disadvantages, as complexity and transferability to humans differ widely. The use of an animal model provides insight into the systemic absorption, distribution, metabolism, and excretion (ADME), but inter-species variation in metabolism may hinder the transferability of data to humans [14].

Xenobiotics are mainly metabolized in the liver by cytochrome P450 (CYP) enzymes. Therefore, many techniques focus on simulating the metabolic activity of the human liver [15]. As oxidative transformation by CYP enzymes is a common pathway for the metabolism of xenobiotics, the electrochemical (EC) conversion of drugs is one of the simplest ways to simulate the metabolism [16, 17]. Different EC cell setups were developed in the past years. Among these, “amperometric” thin-layer cells and “coulometric” flow-through cells are the most commonly used setups for metabolism mimicry. Amperometric thin-layer cells employing working electrode materials such as platinum [18], boron-doped diamond (BDD) [19], or glassy carbon [20] were used to oxidize or reduce a wide range of chemical compounds. However, thin-layer cells only have a small electrode surface area and, therefore, relatively low conversion rates [21]. When using coulometric flow-through cells, both flow and conversion rates can be improved, but electrode fouling may necessitate washing steps of the cell [22]. Göldner et al. developed a coulometric flow-through cell with an exchangeable working electrode to prevent negative effects from fouling and presented a method to generate isotope-labeled acetaminophen in mg scale [23]. As EC oxidation is a purely instrumental method and no substances of human or animal origin are needed, this approach is also favorable from an ethical standpoint. The costs are relatively low and high throughput can be achieved. But the complexity of the system is low and not all oxidation reactions can be mimicked with the EC approach [24, 25].

The use of human subcellular liver fractions (e.g., human liver microsome or S9 fraction) in an in vitro experiment provides insight into the human metabolism without the complexity of using live cells or performing in vivo experiments. The handling of subcellular fractions is straightforward and provides results quickly after a short incubation time [26]. The use of subcellular fractions for the generation of metabolites for anti-doping analysis has been described previously [27–29].

Using living cells provides a more complex model with the possibility of resembling the human in vivo data more closely, yet presents unique challenges in handling [26, 30]. HepaRG™ is a cell line derived from a single female liver tumor patient and can be differentiated into hepatocyte-like cells [31, 32]. HepaRG™ cells have been shown to be a tool for metabolism studies of xenobiotics [33]. Even more complex than 2D cell culture is the use of 3D cell culture in a microfluidic system, a so-called organ on a chip. In this model, organs are represented by spheroids (e.g., liver cells) 100,000 times smaller than human organs [34]. The 3D structure and flow of the cell culture medium resulting in sheer stress on the cells can represent the conditions in vivo better than 2D cell culture [35, 36]. In previous works by Görgens et al., the organ on a chip technology was shown to be able to produce doping-relevant metabolites of the substances stanozolol and DHCMT [37].

The objective of this study is to use EC oxidation, incubation with subcellular human liver fractions, and a 3D culture of HepaRG™ cells in an organ on a chip platform for the generation of metabolites of the SARM RAD140. The samples are analyzed by means of liquid chromatography coupled to high-resolution tandem mass spectrometry (LC-HRMS/MS), and the metabolite patterns produced by the different techniques shall be compared to that of a human urine sample containing RAD140 and its metabolites to evaluate their capability to predict the human metabolism of RAD140.

## Materials and methods

### Chemicals and materials

The reference standard material of RAD140 was purchased from Selleckchem (Houston, TX, USA). Acetonitrile (ACN), ethyl acetate (EtOAc), ammonium formate (NH_4_FA), and Dulbecco’s phosphate-buffered saline (DPBS) solution were purchased from VWR chemicals (Radnor, PA, USA). *Tert*-butyl methyl ether (*t*BME) was acquired from PanReac AppliChem (Darmstadt, Germany). β-Glucuronidase from *E. coli* was obtained from Roche Diagnostics (Mannheim, Germany). Ammonium acetate (NH_4_Ac), Na_2_HPO_4_, nicotinamide adenine dinucleotide phosphate (NADPH), and 3′-phosphoadenosine-5′-phosphosulfate (PAPS) were purchased from Merck (Darmstadt, Germany). Dimethyl sulfoxide (DMSO) was obtained from PAN-Biotech (Aidenbach, Germany). Formic acid (FA) was obtained from Thermo Fisher Scientific (Bremen, Germany). D-Saccharic acid-1,4-lactone (SL), uridine-5′-diphosphoglucuronic acid (UDPGA), KH_2_PO_4_, and MgCl_2_ were acquired from Sigma-Aldrich (St. Louis, MO, USA). Alamethicin was purchased from Enzo Life Sciences (Farmingdale, NY, USA).

Hydrolysis buffer (pH 7) was prepared by combining 8.8 g of NaH_2_PO_4_·H_2_O (Merck) with 17.3 g of Na_2_HPO_4_ (VWR) and adding 230 mL of ultrapure water. Carbonate buffer (pH 10) was prepared by mixing 30 g of K_2_CO_3_ with 30 g of KHCO_3_ (both VWR) and adding 240 mL of ultrapure water. Water was purified using a Barnstead GenPure xCAD Plus from Thermo Scientific.

All samples were combined with the internal standard (IS) prior to workup. The arylpropionamide-derived SARM S-24 was used as IS at a concentration of 100 ng/mL in ACN. S-24 was synthesized in-house according to a previously published procedure [38]. For the generation of analyte/IS peak area ratios, the ion transition *m*/*z* 381.0868 → 241.0594 of the IS was used. All solution compositions are presented as volume to volume ratios.

### Organ on a chip

#### Cell culture

Undifferentiated HepaRG™ cells were obtained from Biopredic International (Rennes, France) and differentiated in-house according to the manufacturer’s instructions and subsequently frozen. HepaRG medium consisted of William’s E medium containing 10% fetal bovine serum, 5 µg/mL human insulin, 5 µg/mL gentamicin sulfate, 0.25 µg/mL amphotericin B (all from PAN-Biotech), 5 × 10^−5^ M hydrocortisone hemisuccinate (Sigma-Aldrich), and 2 mM Glutagrow (Corning, Corning, NY, USA). Human hepatic stellate cells (SteCs) and stellate cell medium were purchased from provitro (Berlin, Germany).

Unless otherwise stated, the cells were cultivated at 37 °C and 5% CO_2_. Four days before the formation of liver spheroids, differentiated HepaRG™ cells were thawed and seeded at 2 × 10^5^ cells/cm^2^ in T75 cell culture flasks. The medium was exchanged 5 h after thawing with medium containing 2% DMSO. The cells were cultured until spheroid formation. Three days before spheroid formation, SteCs (passage 5) were thawed and seeded at 1 × 10^4^ cells/cm^2^ with stellate cell culture medium. The cells were cultured until spheroid formation.

#### Organ on a chip experiment

Four HUMIMIC® Chip2 consisting of two independent microfluidic circuits with two cell compartments in 96-well format each were acquired from TissUse (Berlin, Germany). The pump module used was a HUMIMIC® starter by TissUse, which can run up to four Chip2 simultaneously equaling eight microfluidic circuits. The chips were received filled with DPBS and 0.01% gentamicin/0.1% amphotericin B and prepared by warming them to 37 °C for 24 h before starting pumping with 0.5 Hz and 350 mbar. Three days prior to transfer of the spheroids into the chips, the DPBS was exchanged with HepaRG™ medium and the chips were kept at the same conditions for the rest of the experiment.

The liver spheroids were formed by combining HepaRG™ cells and SteCs in the ratio 25:1 in Eplasia® 96-well plates (Corning) with 79 cavities per well. HepaRG medium, 100 µL, was added into the wells and centrifuged at 200 × g for 1 min to remove air bubbles. The cells were pre-mixed in HepaRG medium, and a total of 2.5 × 10^5^ cells in 100 µL were added to each well. The cells were cultured for 3 days, after which spheroids containing app. 3 × 10^3^ cells each with a diameter of app. 100 µm were formed. Four wells (1 × 10^6^ cells) were combined in a 24-well ultra-low attachment (ULA) plate (Corning) and shaken on an orbital shaker for 5 h. Subsequently, the spheroids were transferred into the chip compartments to give 1 × 10^6^ cells for single spotted circuits and 2 × 10^6^ cells for double spotted circuits. The spheroids were allowed to attach to the compartment bottom for 24 h, after which the pump was started. Two days after the transfer of the spheroids into the chips (d2), a 100% medium exchange was performed with medium containing RAD140 at a concentration of 2.5 µM and 1% DMSO. On d4, d7, d9, d11, d14, d16, and d18, a 50% medium exchange was performed with medium containing RAD140 at a concentration of 2.5 µM and 1% DMSO. A total of eight parallel experiments were run, two circuits each containing 1 × 10^6^ cells with and without the addition of RAD140, one circuit each containing 2 × 10^6^ cells with and without the addition of RAD140, one circuit containing no cells with the addition of RAD140, and one circuit containing 1 × 10^6^ cells without the addition of RAD140 or DMSO.

The exchanged medium was collected for LC-HRMS/MS analysis as well as analysis of cell viability parameters. The experiment was terminated after 21 days. A detailed visualization of the experimental design is shown in the Electronic supplementary material Fig. 3.

#### Cell viability parameters

To determine metabolic activity and cell viability, selected parameters were analyzed. The parameters glucose, lactate dehydrogenase (LDH), and lactate were chosen and monitored during the experiment. All parameters were measured in duplicate; the mean value was used for data evaluation. Glucose was measured with a GlucCell Glucose Monitoring System by Cesco Bioengineering (Taichung, Taiwan). For LDH determination, the LDH Cytotoxicity Assay Kit II from Sigma-Aldrich was used. As positive control, one set of liver spheroids (1 × 10^6^ cells) was lysed in a 1% Triton X-100 solution (Sigma-Aldrich). The supernatant was stored at 4 °C and measured with each set of samples. The samples were diluted fivefold in PBS prior to the measurement; 10 µL of diluted sample was used as input into the assay. Lactate was measured with the Lactate Assay Kit II from Sigma-Aldrich. The samples were diluted fivefold in PBS prior to the measurement; 5 µL of diluted sample was used as input into the assay. The LDH and lactate assays were measured on a Multiskan FC Plate Reader by Thermo Fisher Scientific.

The morphology of the spheroids was monitored by light microscopy using a Primovert microscope from Zeiss (Oberkochen, Germany). A Plan-Achromat 4 × /0.10 objective was used and the photographs were taken with an Axicam 208 color (Zeiss).

#### Sample preparation

The chip supernatants were prepared for LC-HRMS/MS analysis by adding 10 µL IS to 100 µL chip medium. Ice-cold ACN, 400 µL, was added, and the samples were kept on ice for 15 min prior to centrifugation at 17,000 × g for 10 min. The supernatant was reduced in a vacuum centrifuge at 45 °C for 1 h to approximately 100 µL and subsequently extracted twice with 500 µL EtOAc. The organic phases were combined and evaporated at 45 °C for 45 min. The samples were reconstituted in 100 µL of 0.1% FA/ACN (80/20) for LC-HRMS/MS analysis.

### Electrochemical conversion

#### Thin-layer electrochemical cell

A thin-layer µ-PrepCell 2.0 (Antec Scientific, Zoeterwoude, Netherland) was used for the oxidative transformation of RAD140. The cell was equipped with a BDD working electrode, a Pd/H_2_ pseudo reference electrode, and a counter electrode made of conductive polyether ether ketone. An in-house-developed potentiostat and software were used to control the potential. A 2.5 µM solution of RAD140 in 20 mM NH_4_FA buffer (pH 7.4)/ACN (50/50) was oxidized with constant potential at 2100, 2500, and 3200 mV and a flow rate of 20 µL/min. All potentials provided in this work are given relative to the used Pd/H_2_ pseudo reference electrodes.

#### Flow-through electrochemical cell

Additionally, a coulometric flow-through cell developed by Göldner et al. was used [23]. The cell was equipped with a porous graphite working electrode and Pt mesh as counter electrode. As pseudo reference electrode, a Pd wire was used. The potential was controlled with an in-house-developed potentiostat and software. For the oxidation, a 20 µM solution of RAD140 in 20 mM NH_4_FA buffer (pH 7.4)/ACN (50/50) was prepared and oxidized at 1000, 1500, 2000, and 2500 mV. A flow rate of 0.1 mL/min was employed. All potentials provided in this work are given relative to the used Pd/H_2_ pseudo reference electrodes.

#### Sample preparation

Electrochemically oxidized RAD140 solution, 50 µL, was combined with 10 µL IS and 40 µL 0.1% FA/ACN (80/20) for LC-HRMS/MS analysis.

### Subcellular liver fractions

RAD140 was incubated with human subcellular liver fractions to generate metabolites. Human liver microsomes (HLM) and human S9 fractions (S9) (both pooled, 50 mixed-sex donors) were obtained from Thermo Fisher Scientific. Both phase I and consecutive phase I and II experiments were conducted, and all samples were prepared in duplicates. Unless otherwise indicated, all solutions were prepared with in vitro buffer (IB, 50 mM phosphate buffer containing 5 mM MgCl_2_, pH 7.4). A 1-mg/mL solution of RAD140 in DMSO was diluted with IB to reach a final concentration of 100 µM RAD140. To 10 µL of the substrate, 10 µL of a freshly prepared 50 mM NADPH solution was added, as well as 5 µL each of S9 (20 µg/µL) and HLM (20 µg/µL). Two samples lacking subcellular fractions and two samples lacking substrate were prepared to differentiate between enzymatic and non-enzymatic transformation products. To all samples, IB was added until 50 µL total volume was reached. The samples were incubated at 37 °C and 500 rpm for 24 h. To generate phase II metabolites, additional steps were conducted with half of the samples. For this, 10 µL UDPGA (50 mM), 10 µL SL (50 mM), 10 µL PAPS (20 µM), and 10 µL NADPH (50 mM) were added to 2.5 µg of alamethicin. The entire phase I sample and 5 µL each of S9 and HLM were added, and the samples were incubated for an additional 24 h at 37 °C and 500 rpm.

#### Sample preparation

After the incubation, 10 µL IS was added to the samples, followed by 150 µL (phase I) or 300 µL (phase II) of ice-cold ACN. The samples were vortexed and kept on ice for 15 min. Subsequently, the samples were centrifuged at 17,000 × g for 10 min and the supernatant was reduced under vacuum for 60 min at 45 °C. The reduced sample was extracted twice with 500 µL of EtOAc, which was combined and evaporated to dryness. The sample was reconstituted in 0.1% FA/ACN (80/20) for LC-HRMS/MS analysis.

### Human urine sample

The anonymous urine sample analyzed in this study was collected in 2020 during a routine doping control and tested positive for RAD140. No personal information on the individual has been available to the study group. During the sample collection process, the athlete consented to the use of the sample for research purposes. For this project, the sample was measured with and without hydrolysis of the glucuronic acid conjugates.

#### “Dilute-and-shoot”

A total of 50 µL of urine was combined with 10 µL IS and 40 µL 0.1% FA/ACN (80/20) to reach a final dilution of 1:1.

#### Hydrolysis and liquid–liquid extraction

A volume of 200 µL of urine was diluted 1:9 with water, and 10 µL IS was added and combined with 1 mL of hydrolysis buffer and 50 µL of β-glucuronidase. The sample was hydrolyzed at 50 °C for 60 min and subsequently combined with 0.75 mL of carbonate buffer and 5 mL of *t*BME. The sample was extracted for 5 min and centrifuged for 5 min at 1800 × *g*. The organic phase was evaporated and reconstituted in 100 µL 0.1% FA/ACN (80/20) prior to analysis with LC-HRMS/MS.

#### LC-HRMS/MS analysis

The LC-HRMS/MS was conducted on a Vanquish UHPLC system coupled to an Exploris 480 Orbitrap system (both Thermo Scientific). The method used has been previously published [39]. The analytes first detected in this study were added as scheduled PRM experiments. The normalized collision energies (NCE) used for dissociation are listed in Table 1.

**Table 1:** List of detected analytes in the tested human urine sample as well as metabolic and mass spectrometric information

Analyte	Transformation	Molecular formula	RT [min]	Target ions/ion transitions [*m*/*z*]		NCE [%]	Ref
RAD140	–	C_18_H_11_ClN_5_O^−^	6.85	Target ion	348.0658 → 321.0549	20	[4, 39]
				Conf ion 1	348.0658 → 127.0302		
				Conf ion 2	348.0658 → 175.0068		
M1	Hydrolysis Hydroxylation Sulfation	C_8_H_6_ClN_2_O_4_S^−^	3.76	Target ion	260.9742	25	[39, 40]
				Conf ion 1	260.9742 → 181.0174		
				Conf ion 2	260.9742 → 260.9742		
M2a	Hydroxylation Glucuronidation	C_26_H_23_ClN_5_O_9_^−^	4.59	Target ion	584.1190 → 193.0354	30	[39]
				Conf ion 1	584.1190 → 540.0928		
				Conf ion 2	584.1190 → 364.0607		
M2b			4.76	Target ion	584.1190 → 193.0354		[39]
				Conf ion 1	584.1190 → 170.0360		
				Conf ion 2	584.1190 → 390.0763		
M2c^‡^			4.93	Target ion	584.1190 → 364.0607		
				Conf ion 1	584.1190 → 113.0244		
				Conf ion 2	584.1190 → 193.0174		
M3	Hydrolysis Hydroxylation	C_8_H_6_ClN_2_O^−^	5.00	Target ion	181.0174 → 181.0174	50	[39, 40]
				Conf ion 1	181.0174 → 145.0407		
				Conf ion 2	181.0174 → 118.0298		
M4	Glucuronidation	C_26_H_23_ClN_5_O_8_^−^	5.42	Target ion	568.1241 → 193.0354	30	[39, 40]
				Conf ion 1	568.1241 → 113.0244		
				Conf ion 2	568.1241 → 374.0814		
M5	Hydrolysis	C_8_H_8_ClN_2_^+^	5.51	Target ion	167.0371 → 167.0371	50	[39, 40]
				Conf ion 1	167.0371 → 131.0604		
				Conf ion 2	167.0371		
M6a	Hydroxylation	C_18_H_11_ClN_5_O_2_^−^	5.77	Target ion	364.0607 → 170.0360	30	[4, 39]
				Conf ion 1	364.0607 → 193.0174		
				Conf ion 2	364.0607 → 145.0407		
M6b			5.93	Target ion	364.0607 → 170.0360		[4, 39]
				Conf ion 1	364.0607 → 193.0174		
				Conf ion 2	364.0607 → 145.0407		
M6c			6.62	Target ion	364.0607 → 180.0096		[39]
				Conf ion 1	364.0607 → 193.0174		
				Conf ion 2	364.0607 → 145.0407		
M7	Sulfation	C_20_H_15_ClN_5_O_5_S^−^	6.93	Target ion	472.0488	30	[39]
				Conf ion 1	472.0488 → 348.0658		
				Conf ion 2	472.0488 → 96.9601		
M8^‡^	Hydrolysis Hydroxylation Glucuronidation	C_14_H_14_ClN_2_O_7_^−^	3.16	Target ion	357.0495 → 181.0174	30	
				Conf ion 1	357.0495		
				Conf ion 2	157.0495 → 113.0244		
M9^‡^	Dehydrogenation	C_20_H_15_ClN_5_O_2_^+^	5.93	Target ion	392.0909	30	
				Conf ion 1	392.0909 → 177.0214		
				Conf ion 2	392.0909 → 142.0526		
M10a^‡^	Hydroxylation Dehydrogenation	C_18_H_9_ClN_5_O_2_^−^	6.84	Target ion	362.0450 → 145.0407	30	
				Conf ion 1	362.0450 → 362.0450		
				Conf ion 2	362.0450 → 204.0101		
M10b^‡^			7.11	Target ion	362.0450	30	
				Conf ion	362.0450 → 170.0360		
M11^‡^	Loss of water	C_20_H_13_ClN_5_O^−^	7.44	Target ion	374.0814 → 170.0360	30	
				Conf ion 1	374.0814 → 127.0302		
				Conf ion 2	374.0814 → 374.0814		

^‡^Metabolites that are described for the first time. The corresponding HRMS/MS spectra can be found in Electronic supplementary material Figs. 22–27

## Results and discussion

To compare different methods of generating metabolites of RAD140, samples after incubation with subcellular liver fractions, incubation with cultivated HepaRG™-based liver spheroids in an organ on a chip platform, EC conversion, and a human urine sample containing RAD140 were measured with a previously developed and characterized LC-HRMS/MS method and the results compared qualitatively [39]. RAD140 was selected for this study to compare the presented techniques in their capabilities to generate metabolites and expand the knowledge about their applicability for preventive doping research. In addition to already-described metabolites, six new metabolites were detected in this study. These include a hydroxylated and glucuronidated metabolite (M2c), a hydrolyzed, hydroxylated, and glucuronidated metabolite (M8), a dehydrogenated metabolite (M9), two isomers of a hydroxylated and dehydrogenated metabolite (M10a and M10b), and a metabolite after the loss of water (M11). The proposed structures of these metabolites are illustrated in Fig. 1. The HRMS/MS spectra are shown in Electronic supplementary material Figs. 22–27. The structures that were described previously are shown in Electronic supplementary material Fig. 28 [39].

The intact compound RAD140 as well as multiple metabolites are detected in negative ESI mode as the in-source fragment after the loss of acetaldehyde. After RAD140, metabolite M6b showed the longest detection times in micro-dose administration studies [39]. Therefore, it could be of great interest to generate reference material of M6b and implement it into initial testing procedures for sports drug testing. The identity of M5 as 4-amino-2-chloro-3-methylbenzonitrile was confirmed in earlier studies [39, 40]. All detected metabolites are listed in Table 1 with further information regarding their detection. Chromatograms of samples of all investigated techniques are shown in Electronic supplementary material Figs. 4–20.

The newly detected metabolites were identified by searching the mass spectra manually for the specific isotopic pattern of molecules containing one chlorine atom. Subsequently, the detected masses were chosen for MS/MS experiments and specific precursor/product ion transitions were monitored for measurements of the other samples. Not all metabolites shown above were detected in samples of each tested technique. Table 2 summarizes the detected analytes in the measured samples. All chromatograms are additionally shown in the Electronic supplementary material Figs. 4–20. Some metabolites were detected only in trace amounts, i.e., not all ion transitions could be verified. Other metabolites were also detected in the negative control sample containing no subcellular fractions/cells/potential.

**Table 2:** Overview of all detected metabolites and transformation products

	M1	M2a	M2b	M2c^‡^	M3	M4	M5	M6a	M6b	M6c	M7	M8^‡^	M9^‡^	M10a^‡^	M10b^‡^	M11^‡^
Urine hydrolysis and LLE	+	−	−	−	+	−	+	+	+	+	−	−	+	+	+	+
Urine “dilute-and-shoot”	+	+	+	+	+	+	*	+	+	−	+	+	+	−	+	*
Subcellular liver fractions phase I	−	−	−	−	+	−	+	+	+	+	−	−	+	~	~	~
Subcellular liver fractions phase I + II	−	+	−	+	+	+	~	*	+	~	−	+	+	~	~	~
Organ on a chip	+	−	−	*	*	+	~	+	+	~	*	*	+	~	~	~
EC thin-layer cell	−	−	−	−	+	−	+	−	−	+	−	−	+	~	~	~
EC flow-through cell	−	−	−	−	+	−	+	−	−	−	−	−	−	~	~	~

+ detected; − not detected; * detected in trace amounts, i.e., not all diagnostic product ions observed; ~ non-enzymatic transformation, i.e., also detected in samples without subcellular fractions/cells/potential

^‡^Metabolites that are described for the first time

### Human urine sample

**Fig. 2:**
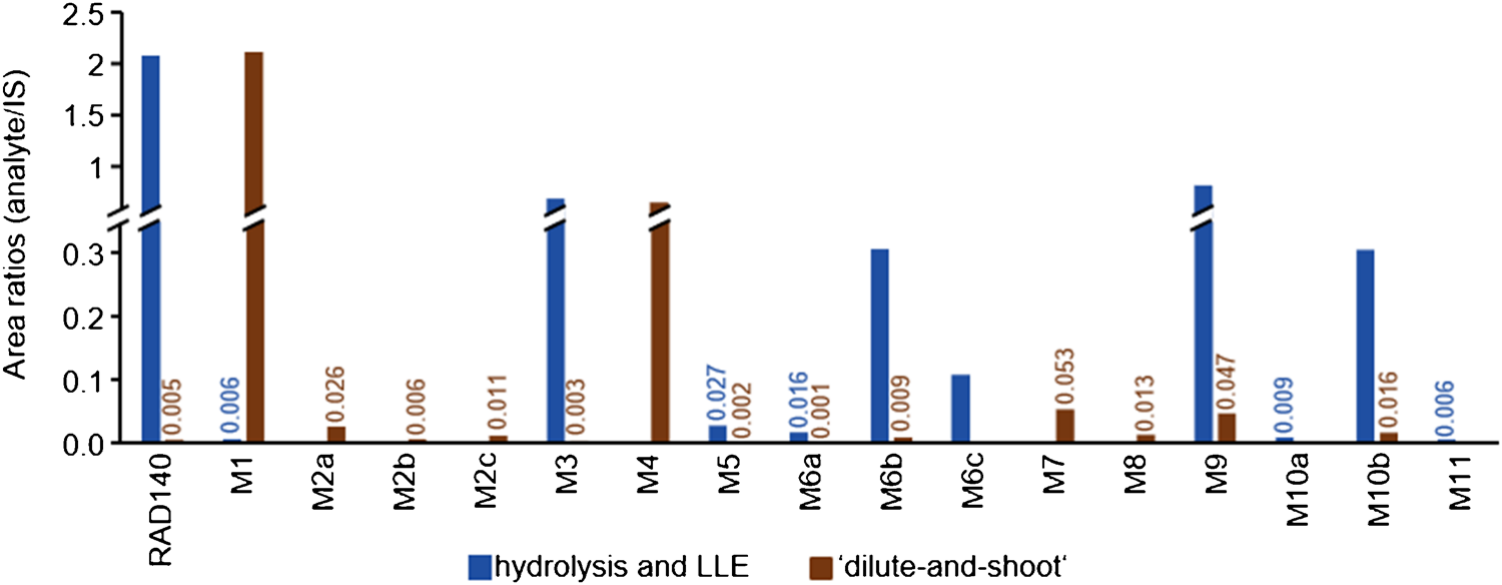
Analyte intensities (peak area ratio analyte/IS) of all detected metabolites in human urine; the intensities of the sample after hydrolysis and LLE are shown in blue, and the intensities of the sample measured with the “dilute-and-shoot” approach are shown in brown. For better visualization, the lower intensities are additionally labeled

An anonymized routine doping control sample was used as a reference sample to estimate the quality of the presented techniques with regard to producing metabolites found in human urine. The urine sample exhibited an estimated concentration of 4 µg/mL RAD140 and was previously used to identify metabolites of RAD140 [39]. It was analyzed both after workup with hydrolysis and liquid–liquid extraction (LLE) and by a “dilute-and-shoot” approach, to ensure the detection of both phase I and phase II metabolites (see “Materials and methods”). Metabolites M1, M3, M5, M6a, M6b, M6c, M9, M10a, M10b, and M11 were detected in the sample after hydrolysis and LLE. Metabolites M1, M2a, M2b, M2c, M3, M4, M5 (trace amount), M6a, M6b, M7, M8, M9, M10b, and M11 (trace amount) were detected in the sample measured by the “dilute-and-shoot” approach. In Fig. 2, the IS-corrected peak areas of the metabolites are shown. As the ionization efficiency may vary between analytes, the relative intensities shall not be confused with the relative concentration of analytes in the sample.

### Organ on a chip

During the organ on a chip experiment, the concentrations of glucose and lactate, as well as cell toxicity (based on LDH levels), were measured to monitor cell viability and metabolic activity. The glucose concentration decreased in all circuits containing cell spheroids, which is a marker for metabolic activity. The spheroids were additionally monitored with light microscopy. Although changes in the morphology of the spheroids could be observed throughout the culture, the cells continued to be metabolically active. Pictures of the cells and more detailed results on cell viability are shown in the Electronic supplementary material Figs. 1–2. The RAD140 concentration of 2.5 µM in the circuits was chosen after stationary cell culture with equivalent spheroids yielded the highest metabolite concentration at this dose (data not shown). A substance application scheme with an application of RAD140 every 2–3 days with a 50% cell culture medium exchange was chosen to simulate a multi-dose application as well as facilitate the detection of long-term metabolites. Two circuits containing 1 × 10^6^ cells each were run as duplicates, as well as one circuit containing 2 × 10^6^ cells. This was done to compare the metabolic activity in dependence on cell amount. As no difference in metabolite intensity based on cell amount could be measured (data not shown), the data from the circuits containing 1 × 10^6^ cells are used. IS was added to the samples prior to the workup for LC-HRMS/MS analysis to reduce the impact of fluctuation during analysis.

**Fig. 3:**
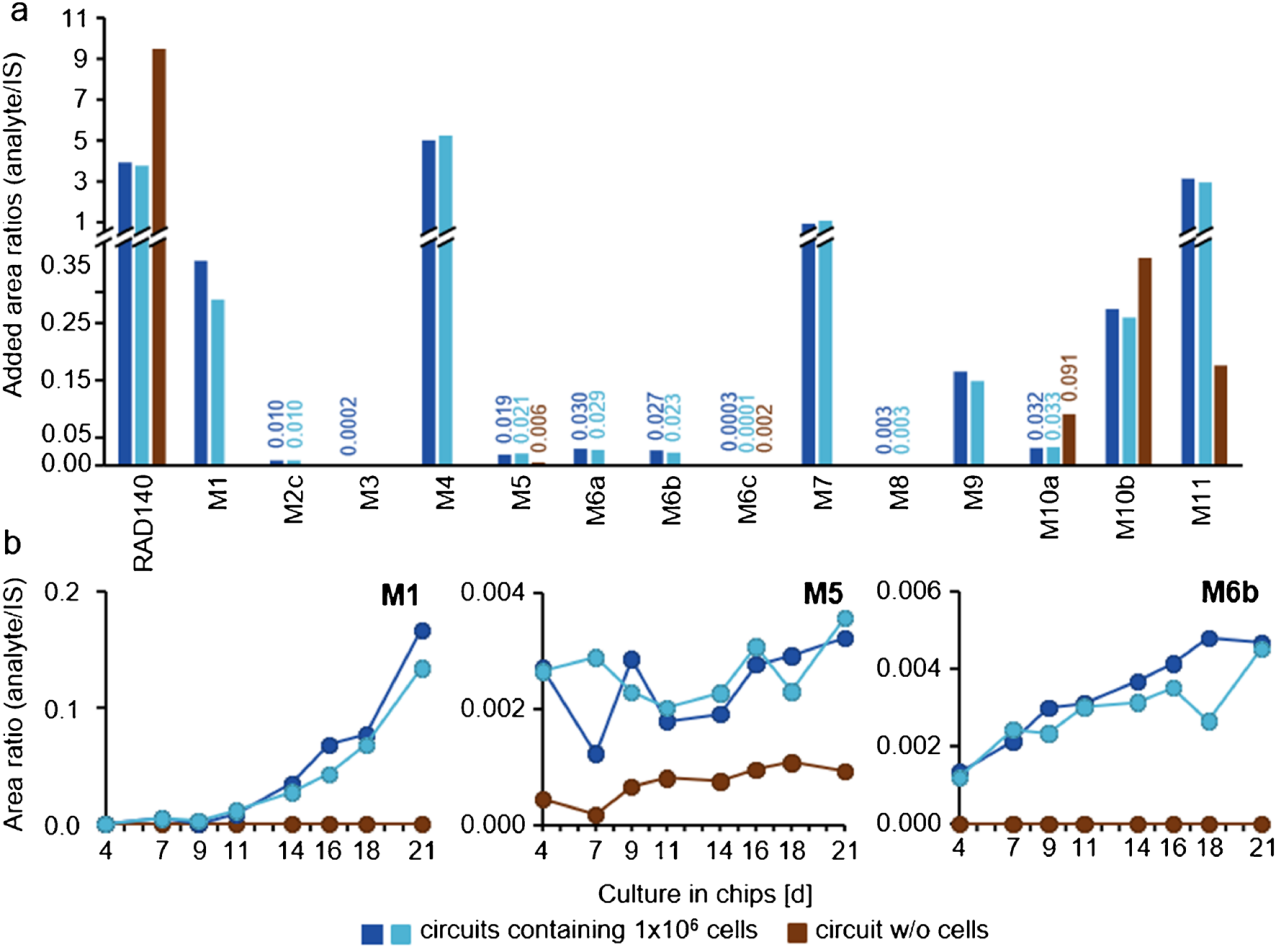
**a** All metabolites that were detected in samples from the organ on a chip experiments are shown as added analyte to IS area ratios from day 4 until day 21 (all samples containing RAD140). Samples from circuits containing 1 × 10^6^ cells run as duplicates are shown in dark blue and light blue; the circuit without cells is shown in brown. **b** Graphs of metabolites M1, M5, and M6b as analyte to IS ratios over the course of the organ on a chip experiment (d4–d21); samples from circuits containing 1 × 10^6^ cells run as duplicates are shown in dark blue and light blue; the circuit without cells is shown in brown. For better visualization, the lower intensities are additionally labeled

Analytes M1, M2c (trace amount), M3 (trace amount), M4, M5, M6a, M6b, M6c, M7 (trace amount), M8 (trace amount), M9, M10a, M10b, and M11 were detected in the organ on a chip samples. The metabolites M5, M6c, M10a, M10b, and M11 were also detected in the circuit that did not contain cell spheroids. This is likely due to non-enzymatic breakdown and/or oxidation of RAD140. For M6c, M10a, and M10b, the concentrations of analyte were higher in the circuits not containing cells compared to the circuits that did contain 1 × 10^6^ cells, which may be due to a lack of competing enzymatic reactions. In Fig. 3a, the intensities of the detected metabolites are shown as analyte to IS ratios (added values of all samples after the application of RAD140, d4-d21). The measured intensity of RAD140 is reduced by about half in the circuits containing cells. Graphs depicting the intensity of three exemplary metabolites in all eight samples that were collected over the course of the organ on a chip experiment are shown in Fig. 3b. M1 and M6b show rising levels of the analyte over the course of the culture. The abundance of M5 does not show a trend over time, which may be due to further metabolization of M5. Analogous graphs for the other metabolites are shown in Electronic supplementary material Fig. 21. None of the detected metabolites show a trend toward lower intensities during the second half of the 21-day culture. Therefore, it can be concluded that even though the morphology of the spheroids changes over the 21-day culture in the chips (see Electronic supplementary material Fig. 1), the metabolic activity remains intact and even results in increased metabolite intensities.

### Subcellular liver fractions

By employing human subcellular liver fractions (HLM and S9) for the metabolism simulation of RAD140, multiple metabolites could be detected. To gain more insight into the metabolism, both phase I and consecutive phase I and II experiments were performed. Detailed information on the experimental design is listed in the “Materials and methods” section.

**Fig. 4:**
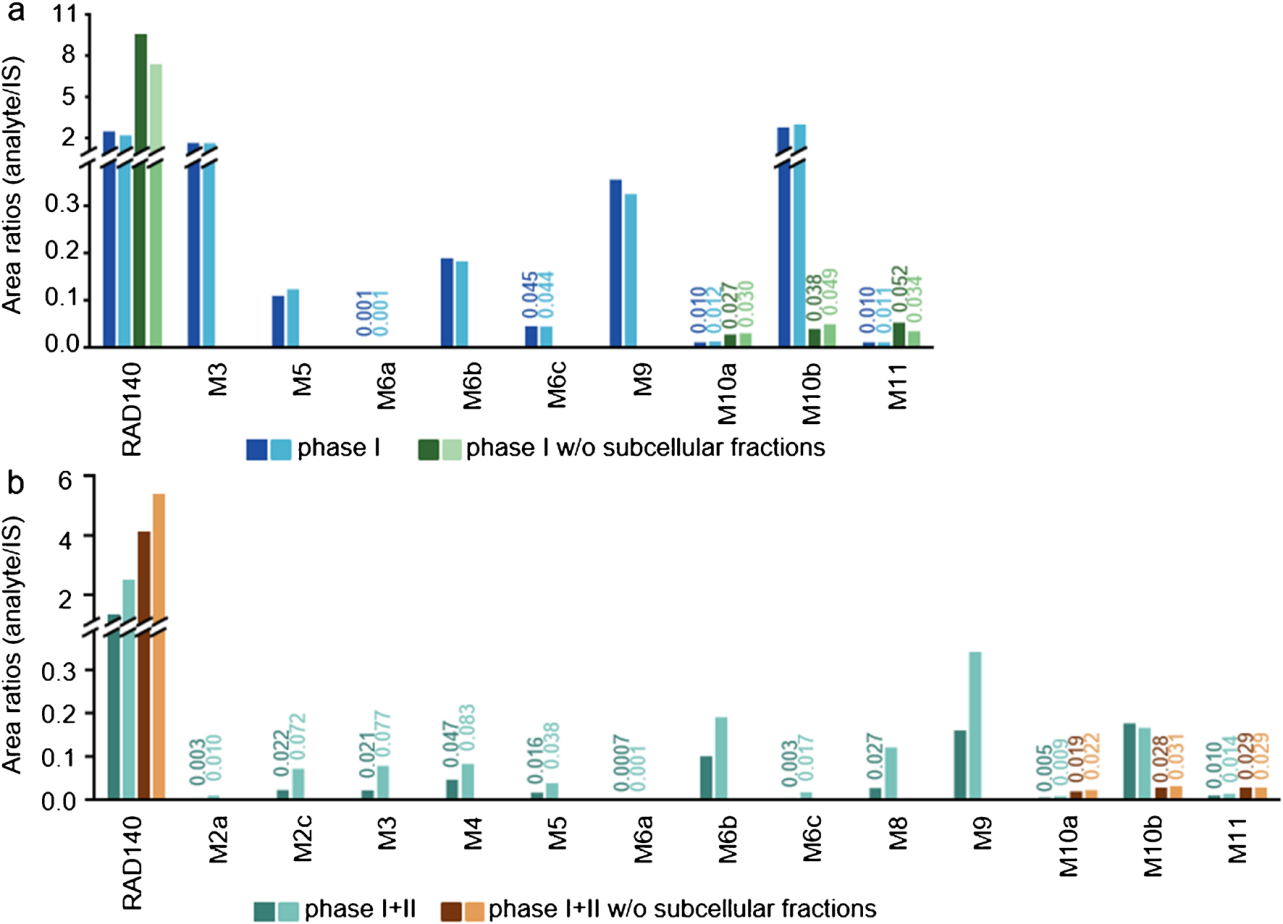
All metabolites that were detected in samples from the incubation with subcellular liver fraction experiments are shown as added analyte to IS area ratios. **a** Samples after phase I incubation are shown in dark blue and light blue, and the corresponding samples lacking the subcellular fractions are shown in dark green and light green. **b** Samples after consecutive phase I + II incubation are shown in dark turquoise and light turquoise, and the corresponding samples lacking the subcellular fractions are shown in dark brown and light brown. For better visualization, the lower intensities are additionally labeled

In Fig. 4, the IS-corrected peak areas of the metabolites are shown, all samples were run as duplicates. After phase I incubation, metabolites M3, M5, M6a, M6b, M6c, M9, M10a, M10b, and M11 were detected in the sample. Yet, M10a, M10b, and M11 were also detectable in the control sample lacking subcellular fractions. After the consecutive phase I and II incubation, in addition to the metabolites detected after phase I incubation, the metabolites M2a, M2c, M4, and M8 were additionally detected in the sample. M6a was detected in trace amount in the sample after phase I and II incubation. The metabolites that show the highest intensity during analysis are M3 and M10b for phase I incubation and M6b and M9 for the consecutive phase I and II incubation. For both M10a and M11, the peak intensities are higher in the samples not containing subcellular fractions. The presence of analytes in the samples not containing subcellular fractions may be due to the non-enzymatic transformation of RAD140 over time, e.g., degradation of RAD140.

### Electrochemical conversion

**Fig. 5:**
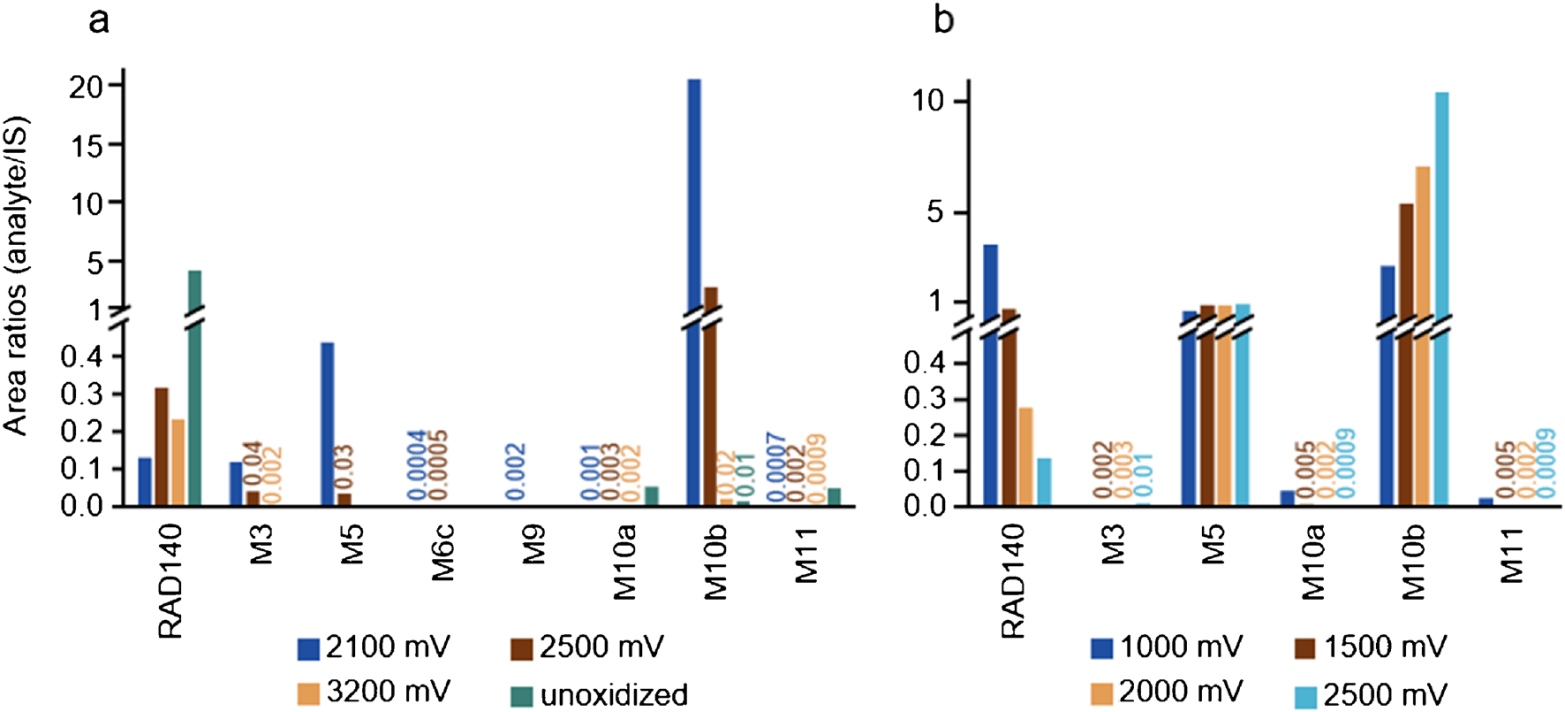
**a** All metabolites that were detected in samples after oxidation in an amperometric thin-layer cell are shown as analyte to IS area ratios. The sample after the oxidation at 2100 mV is shown in dark blue, the sample after the oxidation at 2500 mV is shown in dark brown, the sample after the oxidation at 3200 mV is shown in light brown, and the unoxidized sample is shown in turquoise. **b** All metabolites that were detected in samples after oxidation in a coulometric flow-through cell are shown as analyte to IS area ratios. The sample after the oxidation at 1000 mV is shown in dark blue, the sample after the oxidation at 1500 mV is shown in dark brown, the sample after the oxidation at 2000 mV is shown in light brown, and the sample after the oxidation at 2500 mV is shown in light blue. For better visualization, the lower intensities are additionally labeled

Two different EC cells were tested for the generation of oxidative transformation products of RAD140. In Fig. 5a, the IS-corrected peak areas of the analytes generated in the amperometric thin-layer cell using a BDD working electrode are shown. In Fig. 5b, the IS-corrected peak areas of the analytes generated in a coulometric flow-through cell using a porous carbon working electrode are shown. After oxidation of RAD140 in the thin-layer cell, M3, M5, M6c, M9, M10a, M10b, and M11 were detected. For M3, M5, M9, and M10b, the highest intensity of analyte was detectable after oxidation at 2100 mV. M10a and M11 were detected with the highest intensities in the solution that was not oxidized, which is likely a result of degradation in the used electrolyte. When using the flow-through cell, M3, M5, M10a, M10b, and M11 were detectable. M3, M5, and M10b showed the highest intensities after oxidation at 2500 mV. M10a and M11 show the highest intensities after oxidation at 1000 mV; however, this is likely also a result of degradation in the electrolyte. An increase of the flow rate to 0.5 and 1.0 mL/min did not increase the intensity of the formed analytes in the flow-through cell (data not shown).

### Comparison of in vitro techniques

The urine sample contained 16 metabolites that were detected with varying intensities. As this is a single spot urine of a male athlete providing no information on the time and dose of RAD140 intake, the metabolic profile cannot be generalized for all urine samples after the intake of RAD140.

Six novel metabolites of RAD140 were identified by analysis of mass spectrometric data of samples after in vitro incubation experiments. All six metabolites were also detected in the analyzed doping control urine sample. As the background signals in the mass spectrometric analysis can impede metabolite identification, the lower matrix load in in vitro samples compared to urine samples simplified the identification of metabolites. All tested approaches for the generation of metabolites of RAD140 resulted in detectable amounts of transformation products. However, not all techniques yielded the same products. The organ on a chip technology using 3D HepaRG™-based liver spheroids resulted in the highest number of detected metabolites (14 out of 16), however, four of those metabolites could only be detected in trace amounts, meaning that not all monitored ion transitions were verifiable in the samples. The metabolite M1 was only detected in the organ on a chip samples. The approaches using human subcellular liver fractions resulted in 13 detected metabolites. The metabolite M2a was only detected in the samples after incubation with subcellular liver fractions.

The organ on a chip platform allows for the culture of living cells in a micro-fluidic environment, which resembles the conditions in the human organism more closely than isolated subcellular fractions. The used HepaRG™ cells are a well-understood in vitro model to generate drug metabolites [31, 41, 42]; however, the 3D culture in a microfluidic environment is a novel technology. In this system, experiments such as multi-dose application and culture over several weeks are possible to facilitate the generation of long-term metabolites. But drug concentration levels are limited when working with living cells, as cell toxicity effects can influence the cell physiology and impede metabolism. The initial concentration of RAD140 was 20 µM and 10 µM for phase I incubation and phase II incubation with subcellular liver fractions, respectively. This represents eight- and four-times more concentrated situations compared to the organ on a chip experiments, respectively. This may also lead to higher metabolite concentrations, i.e., superior detectability by mass spectrometric analysis. An additional advantage of the subcellular liver fractions is the use of pooled fractions of 50 donors that were used for the experiments. This approach reduces the impact of inter-individual variation in enzyme expression and activity, as opposed to the HepaRG™ cells used for the organ on a chip experiment, which is a cell line derived from a single female donor. The sample preparation for the subcellular fraction and organ on a chip samples employed an extraction with EtOAc to ensure extraction of both phase I and phase II metabolites. Because of the higher volume available regarding the doping control urine sample, the sample was measured both after hydrolysis and LLE and a dilute-and-shoot approach to detect phase I and phase II metabolites separately. This may influence the relative intensities of the metabolites.

The EC approach resulted in the lowest number of metabolites with 7 and 5 detectable in the samples of the amperometric thin-layer cell and coulometric flow-through cell, respectively. As the EC conversion is purely instrumental and a far less complex system compared to the other presented techniques, the reduced number of detected metabolites was expected. However, due to its reduced complexity, EC is easily established in laboratories. The metabolites that may be generated in sufficient yield for semipreparative synthesis and isolation using the coulometric flow-through cell are M5 and M10b.

The urinary metabolite M2b could not be detected in any of the metabolism simulation samples analyzed during this study. Some metabolites were only detected in one of the tested techniques, namely M1 in the organ on a chip samples and M2a in the samples after consecutive phase I and II incubation with subcellular liver fractions. Therefore, the organ on a chip approach used in this project and the incubation with subcellular liver fractions can be viewed as complementary to each other, as none of the approaches could produce all metabolites found in human urine.

Metabolite M6b, which showed the longest detection time after the parent compound in previous micro-dose studies [39], could only be detected with low intensity in the measured samples. The technique which may facilitate the generation of mg amounts of metabolites, the coulometric flow-through cell, did not show the generation of M6b. Therefore, a conventional synthesis of the metabolite may be an objective for future projects. Alternatively, extracts from subcellular fraction or organ on a chip experiments may be added to urine samples as positive control samples for doping control testing procedures as long as no synthesized standard material is available.

Although some detected analytes are most likely resulting from non-enzymatic transformation, they may nonetheless be of value for anti-doping analysis, as they are also detectable in the human urine sample. The relevance of the newly detected metabolites M2c, M8, M9, M10a, M10b, and M11 for doping control purposes has to be further investigated as no data from controlled human administration studies is available on the excretion behavior and detection times of these metabolites. The results for the metabolism simulation of RAD140 are not necessarily transferable to other doping-relevant substances. More studies on the application of the EC and organ on a chip method are therefore important, to further increase the knowledge on the metabolic conversion of substances for which no human in vivo data are available so far.

## Conclusion

The goals of this study were to investigate the metabolism of and compare different in vitro techniques to produce metabolites of the WADA-prohibited anabolic agent RAD140. This knowledge may improve insight into the applicability of the organ on a chip technology and EC techniques in doping control analysis.

Multiple metabolites and transformation products of the SARM RAD140 were produced by HepaRG™-based liver spheroids in the organ on a chip platform, incubation with subcellular liver fractions, and EC oxidation. The comparison of the techniques showed that the organ on a chip approach yielded the highest number of detected metabolites, but some of the analytes were only detected in trace amounts. The subcellular liver fractions yielded the second-highest number of metabolites, and the EC approach using two different EC cells yielded the smallest number of detectable metabolites. Two metabolites were detected with high intensities using EC that may facilitate the semipreparative synthesis and isolation of sufficient amounts for use as reference materials for further studies or for structural elucidation using NMR spectroscopy. In conclusion, both organ on a chip with HepaRG™ liver spheroids and subcellular liver fractions produced a majority of metabolites found in the human urine sample and each technique yielded one metabolite not produced by the other technique. Both can therefore be seen as complementary to one another for the generation of human metabolites of RAD140.

The expansion of techniques to generate biotransformation products may help in deepening the knowledge about the metabolism of novel doping substances and yield possible targets for routine anti-doping analysis. As SARMs rise in popularity, comprehensive information on biotransformation and excretion behavior is necessary to detect doping. In the future, organ on a chip experiments combining multiple organoids, such as skin-liver and liver-kidney combinations, may provide more predictive power as the simulation of ADME can be improved.

## Supplementary Information

Below is the link to the electronic supplementary material.

Supplementary file1 (PDF 1136 KB)

## Data Availability

The datasets generated and analyzed during the current study are available from the corresponding author on reasonable request.
