# Supplementary material for: Comparison of in vitro approaches for predicting the metabolism of the selective androgen receptor modulator RAD140
Source: Anal Bioanal Chem. 2023 Jul 8;415(23):5657–69. doi: 10.1007/s00216-023-04835-z (PMC10473985; doi:10.1007/s00216-023-04835-z)
Supplement: Supplementary file 1 — Supplementary file1 (PDF 1136 KB) [file 216_2023_4835_MOESM1_ESM.pdf]

Electronic Supplementary Material

Comparison of *In Vitro* Approaches for Predicting the Metabolism of the Selective Androgen Receptor Modulator RAD140

Felicitas Wagener[1], Nana Naumann[1], Valentin Göldner[2,3], Christian Görgens[1], Sven Guddat[1], Uwe Karst[2,3], Mario Thevis*[,1,4]

[1] Institute of Biochemistry, German Sport University Cologne, Cologne, Germany

[2] Institute of Inorganic and Analytical Chemistry, University of Münster, Münster, Germany

[3] International Graduate School for Battery Chemistry, Characterization, Analysis, Recycling and Application (BACCARA), University of Münster, Münster, Germany

[4] European Monitoring Center for Emerging Doping Agents (EuMoCEDA), Cologne, Germany

* ORCID ID 0000-0002-1535-6451

Corresponding author: Mario Thevis, PhD, Center for Preventive Doping Research/Institute of Biochemistry, German Sport University Cologne, Am Sportpark Müngersdorf 6, 50933 Cologne, Germany, Tel.: +49 221 4982 7070, m.thevis@biochem.dshs-koeln.de

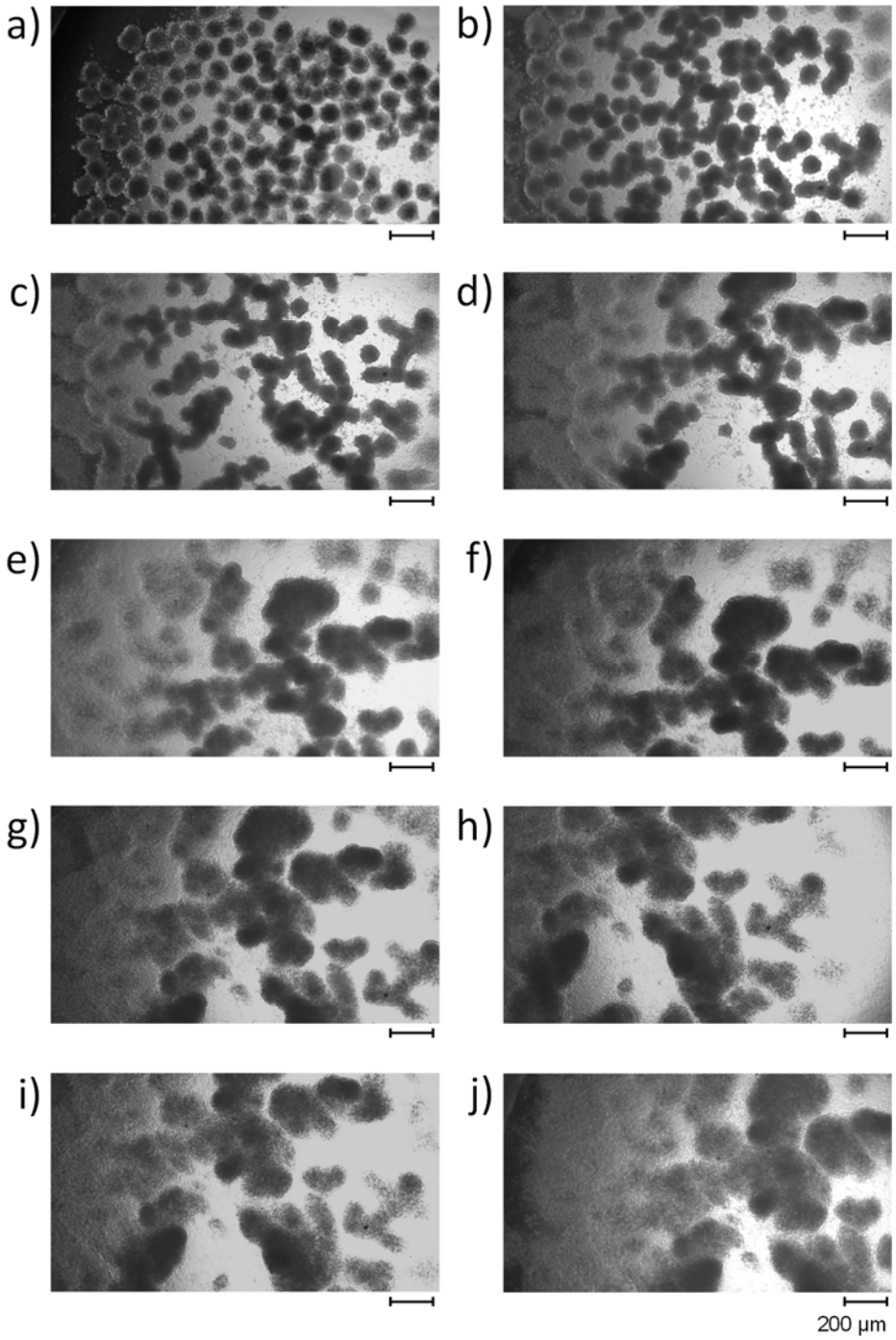

Supplementary Figure 1: Microscopic pictures of the liver spheroids in the chip compartment. The pictures show an exemplary circuit containing $1x10^6$ cells, 2.5 µM RAD140 and 1% DMSO. a) d0 b) d2 c) d4 d) d7 e) d9 f) d11 g) d14 h) d16 i) d18 j) d21. Color and contrast of the images was adjusted for better visibility.

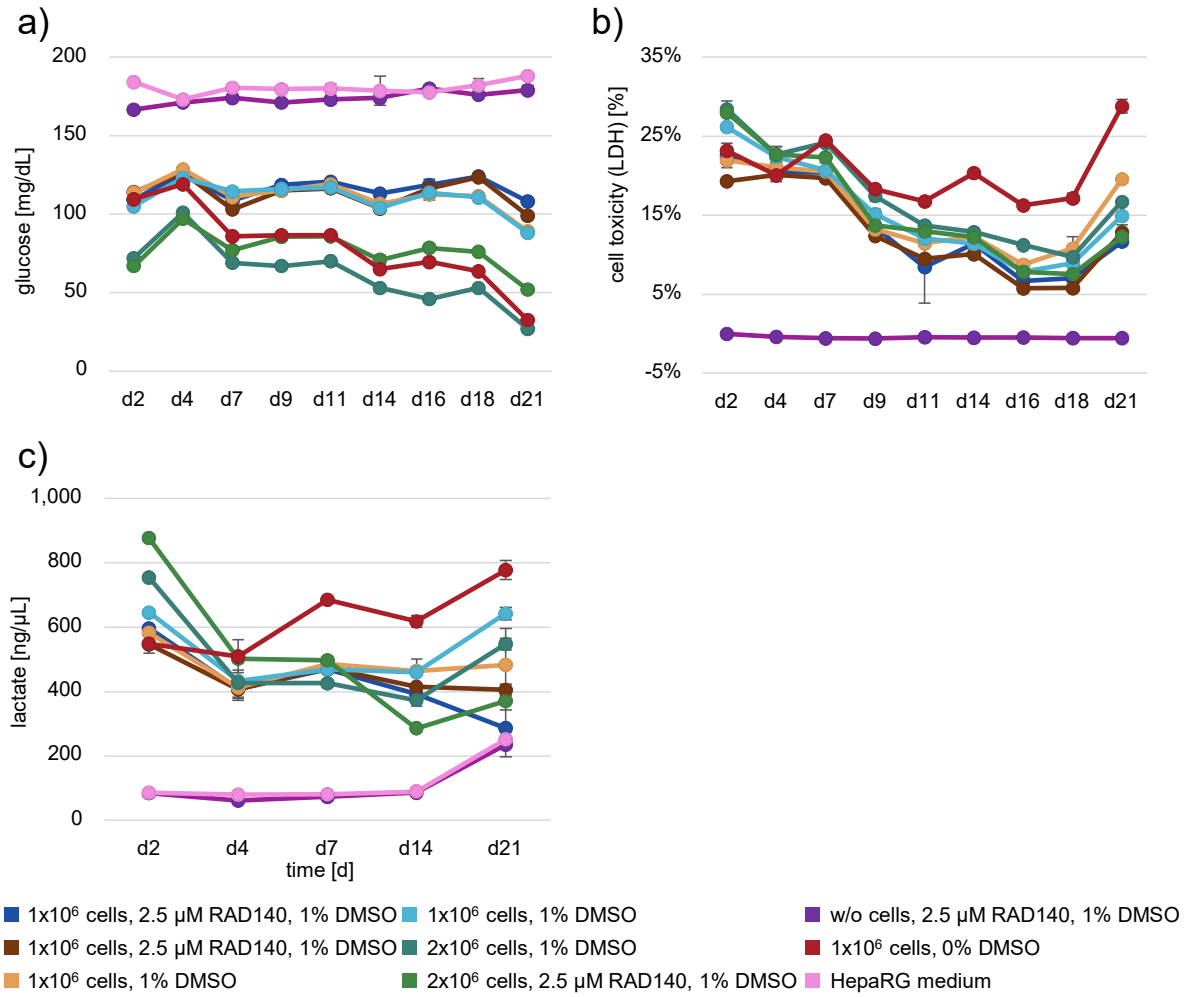

Supplementary Figure 2: cell viability parameters measured during the chip experiments. The samples were measured in duplicates and error bars show the standard deviation. a) glucose concentration b) cell toxicity determined from LDH measurements c) lactate concentration.

All circuits containing cells showed reduced glucose concentrations compared to fresh medium and the circuit without cells. This indicated that the cells were metabolically active during the whole experiment. As expected, circuits with cells in both compartments showed higher glucose consumption compared to circuits with cells in one compartment. Additionally, the circuit in which no DMSO was added, the glucose consumption was also elevated. This might indicate inhibition of metabolic activity. Therefore, in future experiments, the DMSO concentration should be further reduced. The cell toxicity was determined by assessing LDH levels relatively to lysed cells (100% cell toxicity) and medium (0% cell toxicity). LDH levels indicted cytotoxicity between 19-29% at the beginning of the cultivation in the chips, which fell over time to 8-14% on day 11 and rose towards the end of the experiment to 12-29%. Lactate concentration was higher in circuits containing cells than in the medium control. Lactate levels in circuits containing cells started between 500-900 ng/µL and fell during the experiment to around 300-600 ng/µL and rose slightly towards the end of the experiment. The circuit with the highest cell toxicity and the highest lactate concentration was the one without the addition of DMSO and RAD140.

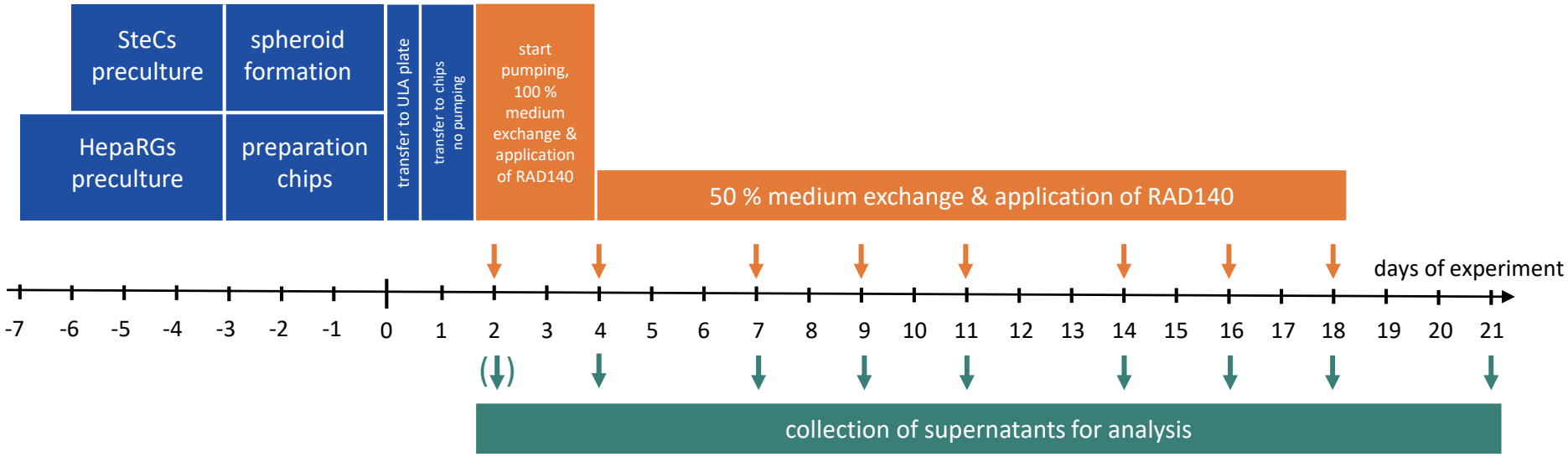

Supplementary Figure 3: Experimental design of RAD140 organ-on-a-chip experiment. Counting of days begins at spheroid transfer in ULA plate. Orange and green arrows indicate the exchange of 200 µL medium containing 1 µg/mL RAD140 and 1% DMSO.

RAD140

**target peak**	*m/z* 348.0658->321.0549
**conf peak 1**	*m/z* 348.0658->127.0302
**conf peak 2**	*m/z* 348.0658->175.0068

doping control urine hydrolysis+LLE

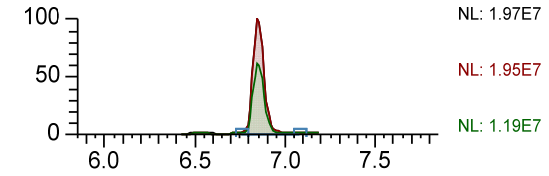

EC flow-through cell 2000 mV

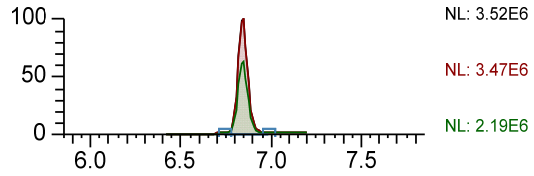

doping control urine 'dilute and shoot'

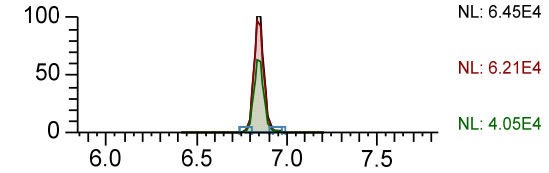

EC thin-layer cell 2100 mV

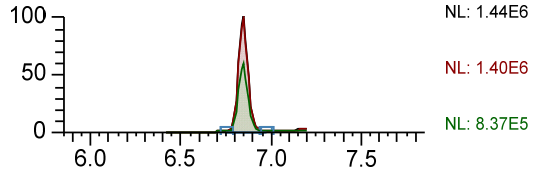

subcellular liver fractions phase I

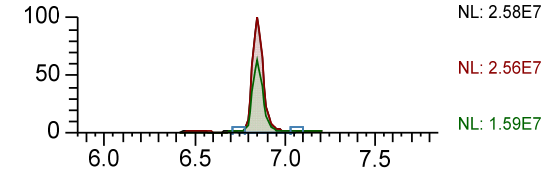

EC unoxidized

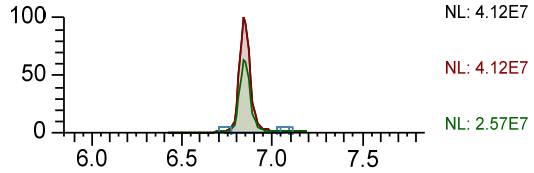

subcellular liver fractions phase I w/o fractions

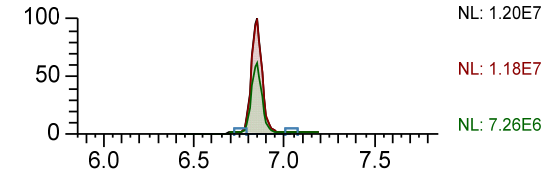

organ-on-a-chip $1 \cdot 10^6$ cells d14

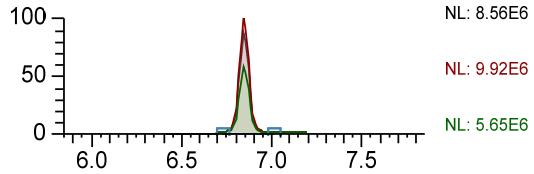

subcellular liver fractions phase I+II

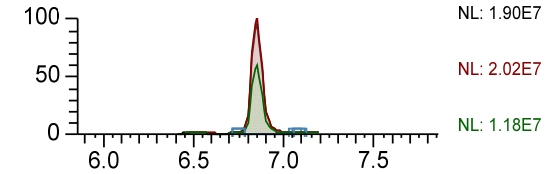

organ-on-a-chip w/o cells d14

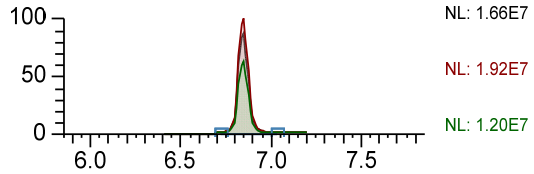

subcellular liver fractions phase I+II w/o fractions

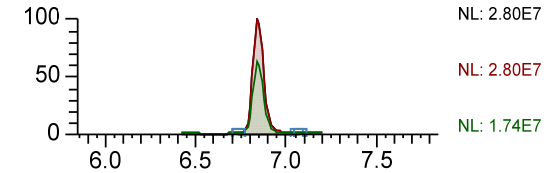

RAD140 standard

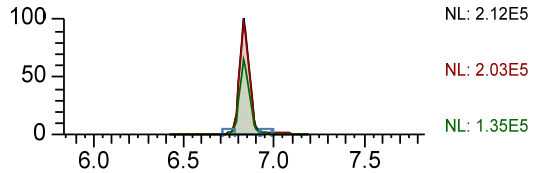

Supplementary Figure 4: Exemplary chromatograms of RAD140 in the measured samples.

M1

**target peak**	*m/z* 260.9742
**conf peak 1**	*m/z* 260.9742->181.0174
**conf peak 2**	*m/z* 260.9742->260.9742

doping control urine hydrolysis+LLE

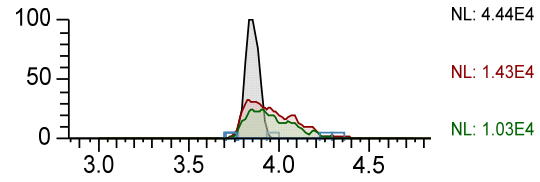

EC flow-through cell 2000 mV

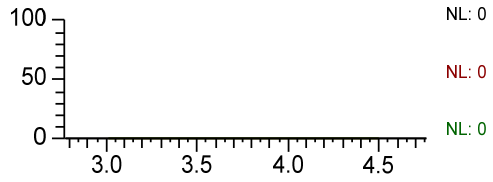

doping control urine 'dilute and shoot'

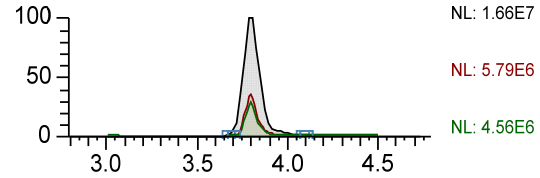

EC thin-layer cell 2100 mV

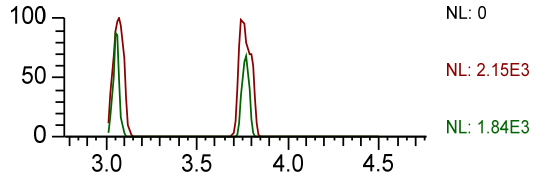

subcellular liver fractions phase I

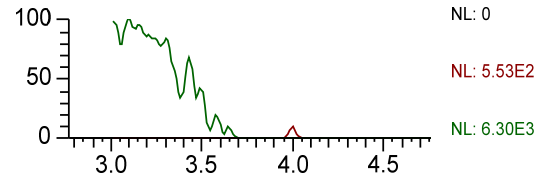

EC unoxidized

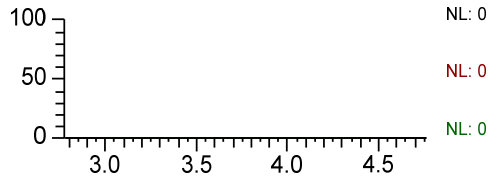

subcellular liver fractions phase I w/o fractions

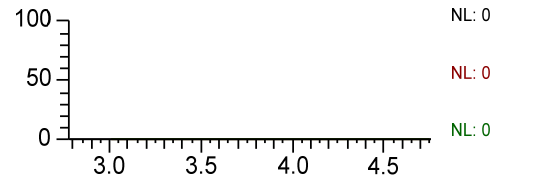

organ-on-a-chip 1·10^6 cells d14

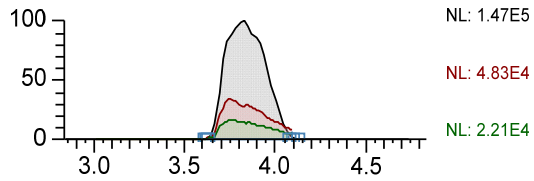

subcellular liver fractions phase I+II

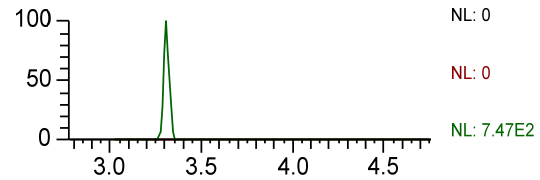

organ-on-a-chip w/o cells d14

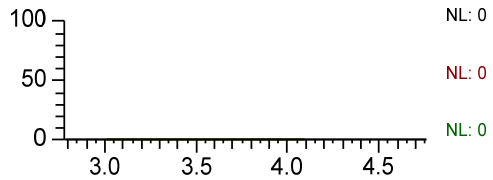

subcellular liver fractions phase I+II w/o fractions

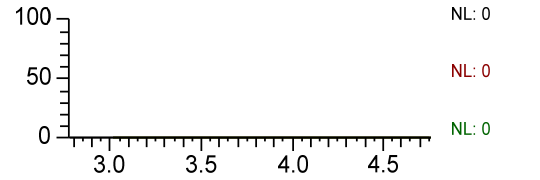

RAD140 standard

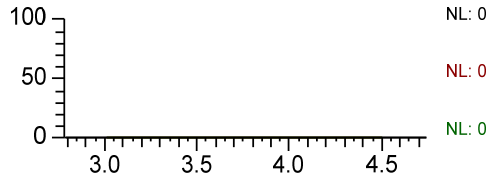

Supplementary Figure 5: Exemplary chromatograms of M1 in the measured samples.

M2a

**target peak**	*m/z* 584.1190->193.0354
**conf peak 1**	*m/z* 584.1190->540.0928
**conf peak 2**	*m/z* 584.1190->364.0607

doping control urine hydrolysis+LLE

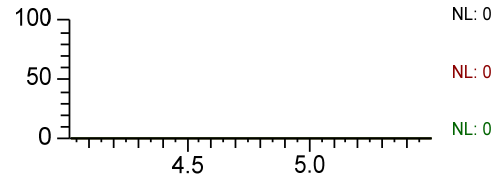

EC flow-through cell 2000 mV

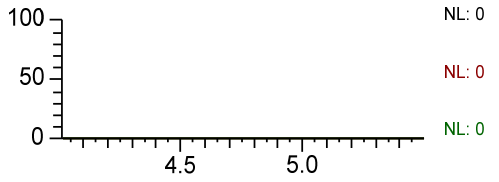

doping control urine 'dilute and shoot'

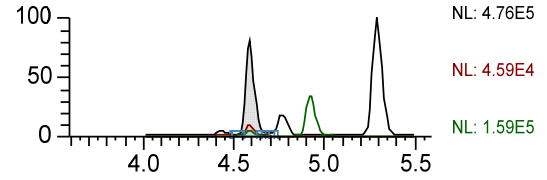

EC thin-layer cell 2100 mV

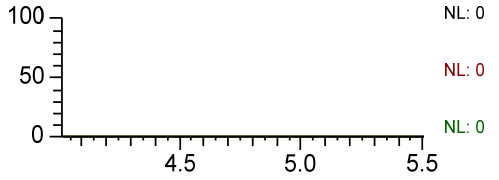

subcellular liver fractions phase I

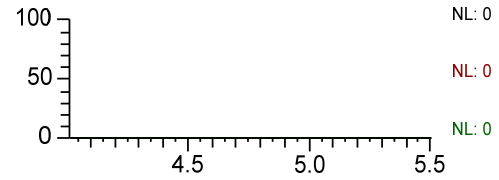

EC unoxidized

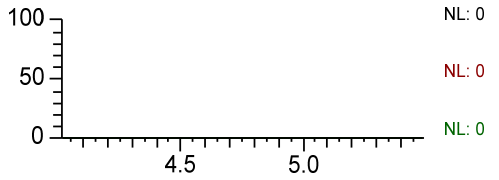

subcellular liver fractions phase I w/o fractions

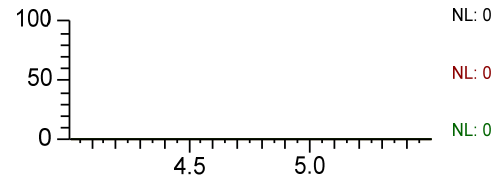

organ-on-a-chip $1 \cdot 10^6$ cells d14

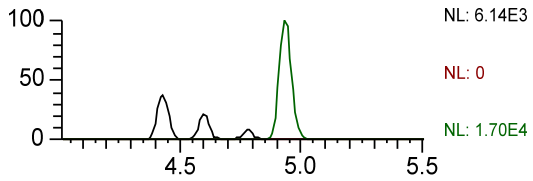

subcellular liver fractions phase I+II

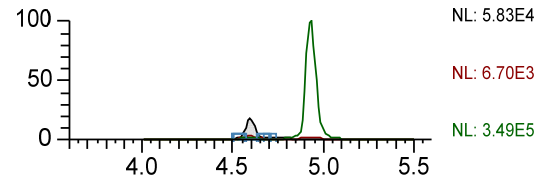

organ-on-a-chip w/o cells d14

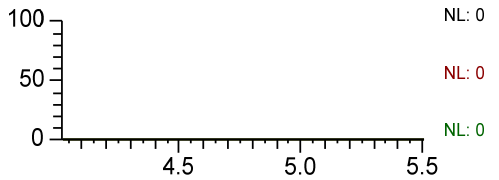

subcellular liver fractions phase I+II w/o fractions

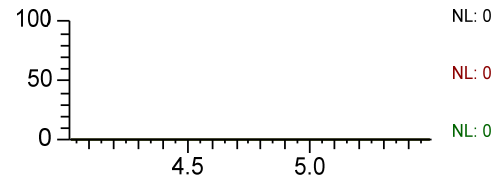

RAD140 standard

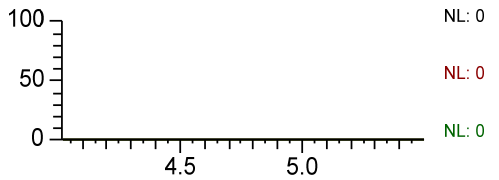

Supplementary Figure 6: Exemplary chromatograms of M2a in the measured samples.

M2b

**target peak**	*m/z* 584.1190->193.0354
**conf peak 1**	*m/z* 584.1190->170.0360
**conf peak 2**	*m/z* 584.1190->390.0763

doping control urine hydrolysis+LLE

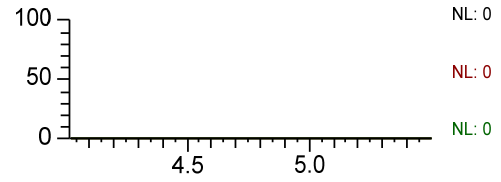

EC flow-through cell 2000 mV

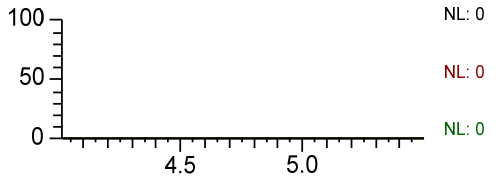

doping control urine 'dilute and shoot'

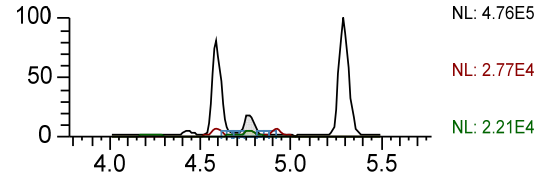

EC thin-layer cell 2100 mV

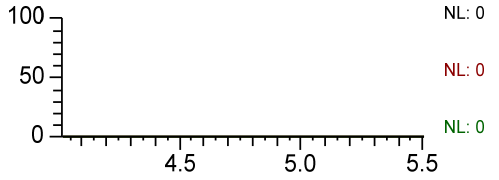

subcellular liver fractions phase I

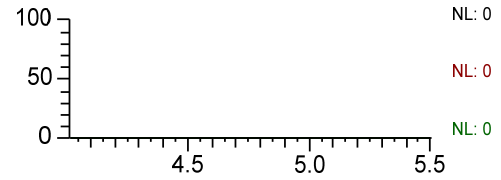

EC unoxidized

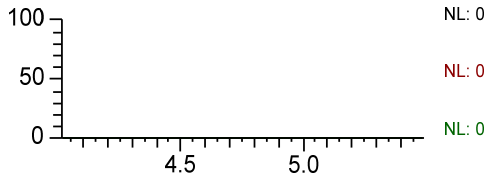

subcellular liver fractions phase I w/o fractions

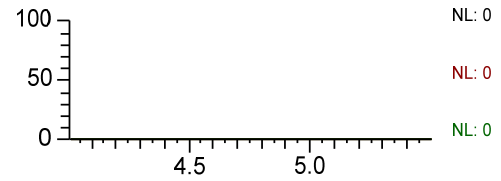

organ-on-a-chip $1 \cdot 10^6$ cells d14

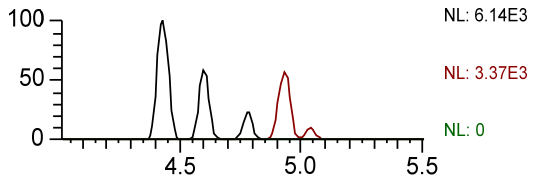

subcellular liver fractions phase I+II

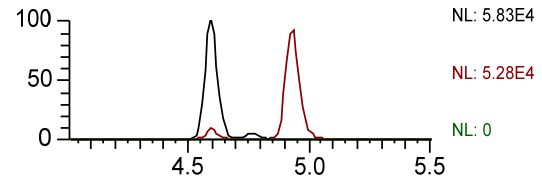

organ-on-a-chip w/o cells d14

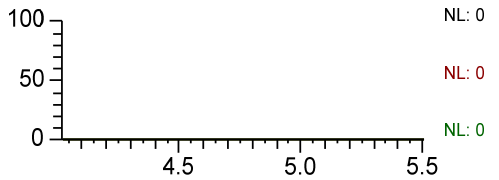

subcellular liver fractions phase I+II w/o fractions

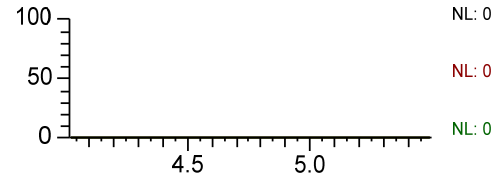

RAD140 standard

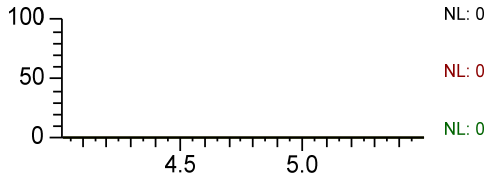

Supplementary Figure 7: Exemplary chromatograms of M2b in the measured samples.

M2c

**target peak**	*m/z* 584.1190->364.0607
**conf peak 1**	*m/z* 584.1190->113.0244
**conf peak 2**	*m/z* 584.1190->193.0174

doping control urine hydrolysis+LLE

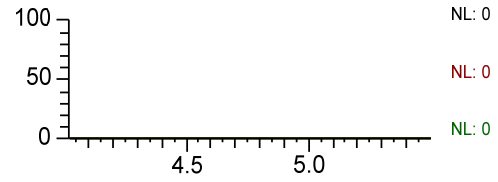

EC flow-through cell 2000 mV

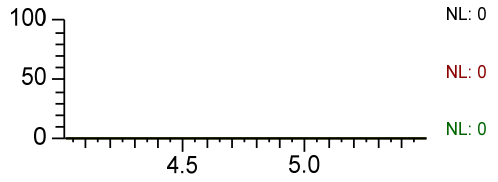

doping control urine 'dilute and shoot'

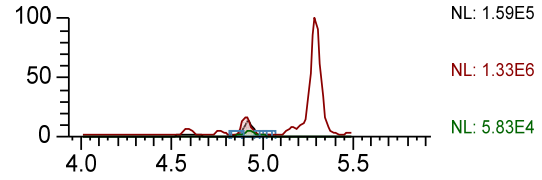

EC thin-layer cell 2100 mV

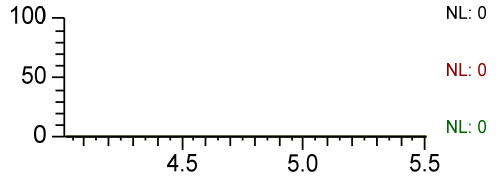

subcellular liver fractions phase I

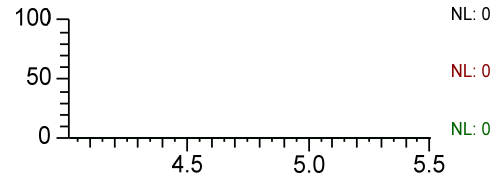

EC unoxidized

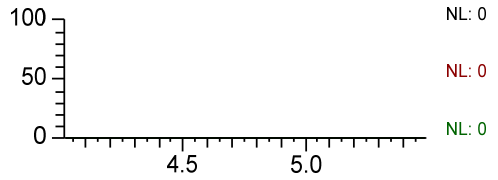

subcellular liver fractions phase I w/o fractions

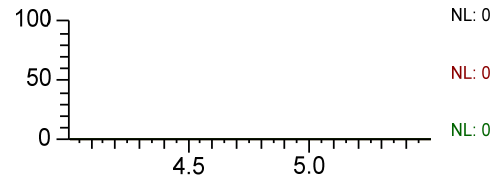

organ-on-a-chip $1 \cdot 10^6$ cells d14

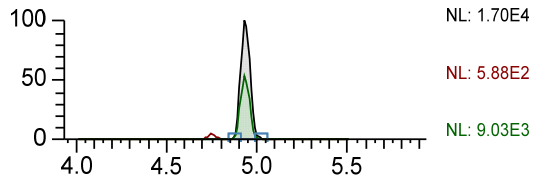

subcellular liver fractions phase I+II

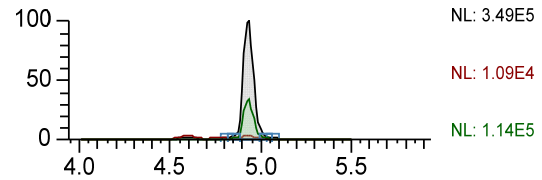

organ-on-a-chip w/o cells d14

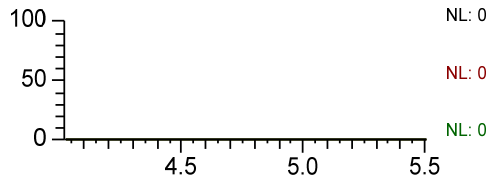

subcellular liver fractions phase I+II w/o fractions

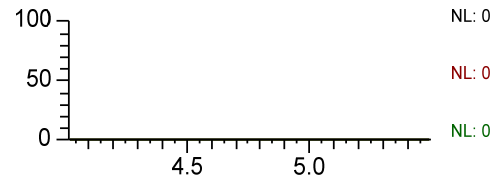

RAD140 standard

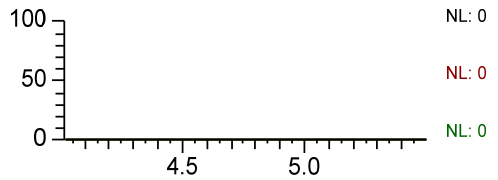

Supplementary Figure 8: Exemplary chromatograms of M2c in the measured samples.

M3

**target peak**	*m/z* 181.0174->181.0174
**conf peak 1**	*m/z* 181.0174->145.0407
**conf peak 2**	*m/z* 181.0174->118.0298

doping control urine hydrolysis+LLE

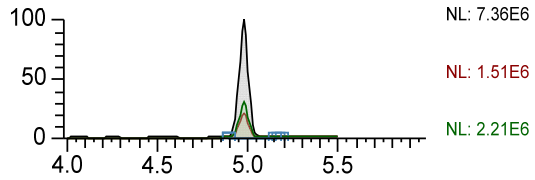

EC flow-through cell 2000 mV

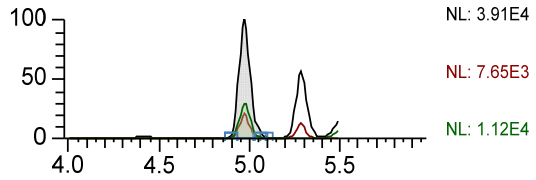

doping control urine 'dilute and shoot'

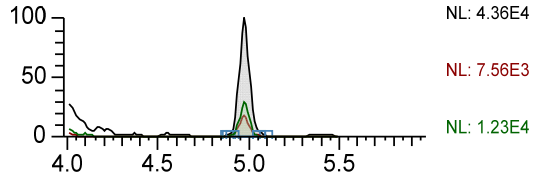

EC thin-layer cell 2100 mV

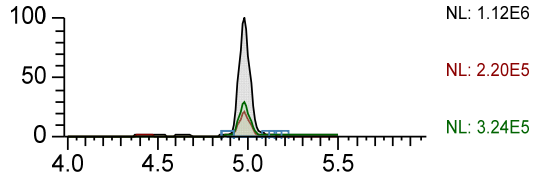

subcellular liver fractions phase I

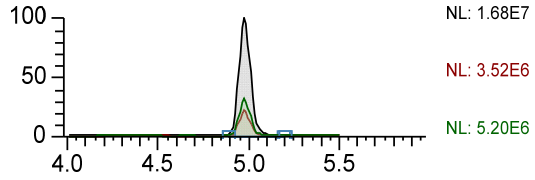

EC unoxidized

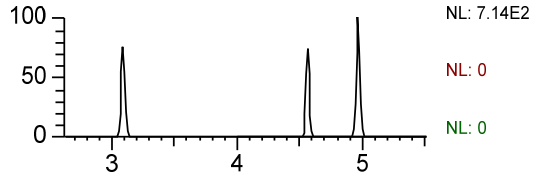

subcellular liver fractions phase I w/o fractions

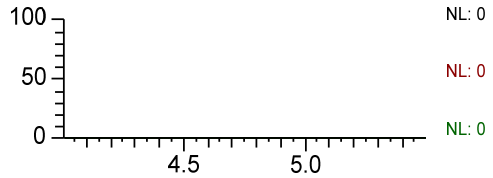

organ-on-a-chip 1·10^6 cells d14

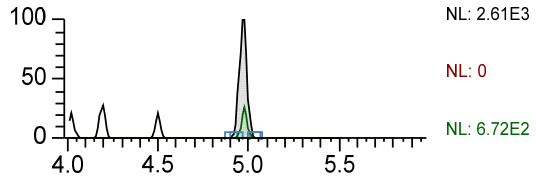

subcellular liver fractions phase I+II

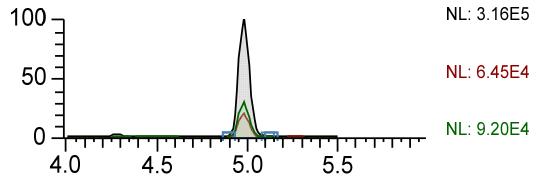

organ-on-a-chip w/o cells d14

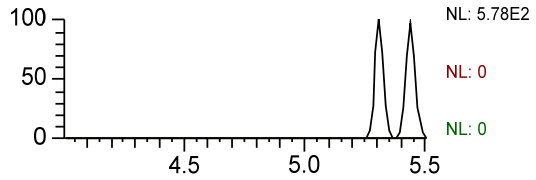

subcellular liver fractions phase I+II w/o fractions

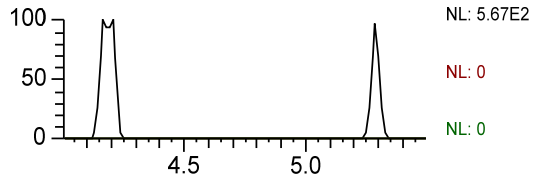

RAD140 standard

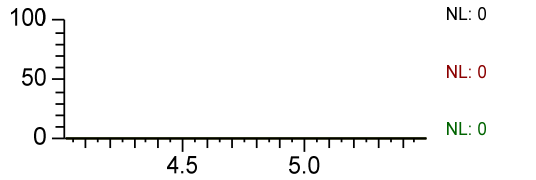

Supplementary Figure 9: Exemplary chromatograms of M3 in the measured samples.

M4

**target peak**	*m/z* 568.1241->193.0354
**conf peak 1**	*m/z* 568.1241->113.0244
**conf peak 2**	*m/z* 568.1241->374.0814

doping control urine hydrolysis+LLE

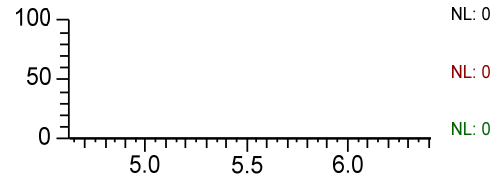

EC flow-through cell 2000 mV

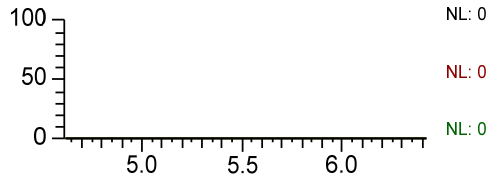

doping control urine 'dilute and shoot'

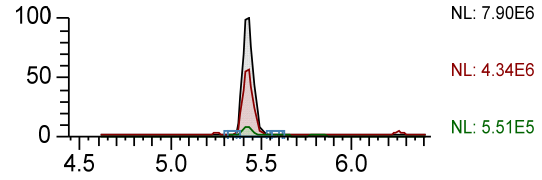

EC thin-layer cell 2100 mV

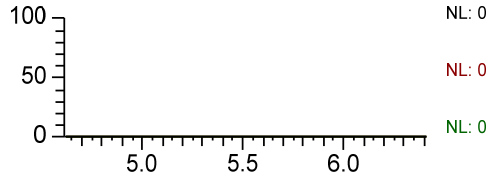

subcellular liver fractions phase I

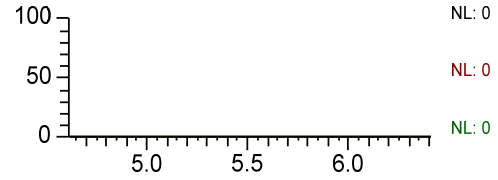

EC unoxidized

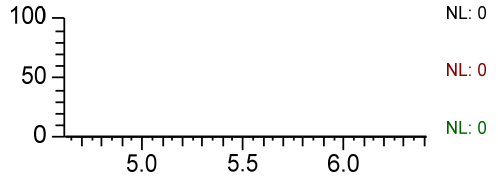

subcellular liver fractions phase I w/o fractions

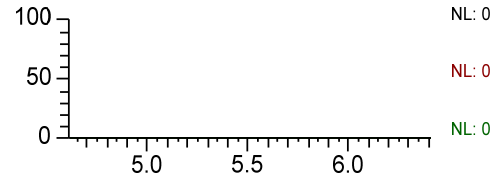

organ-on-a-chip $1 \cdot 10^6$ cells d14

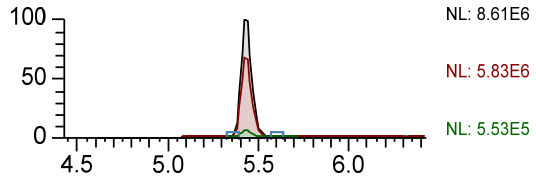

subcellular liver fractions phase I+II

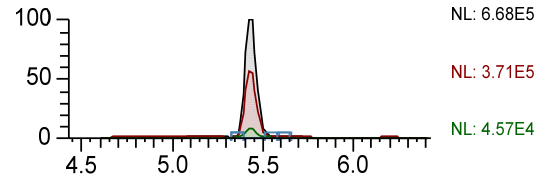

organ-on-a-chip w/o cells d14

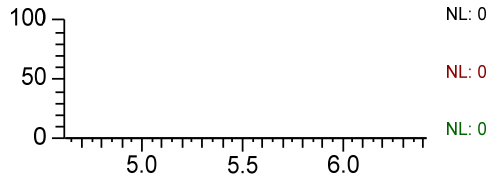

subcellular liver fractions phase I+II w/o fractions

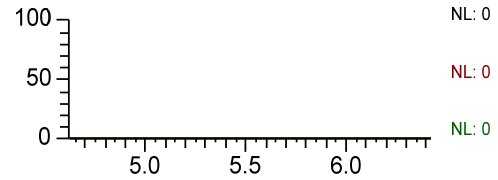

RAD140 standard

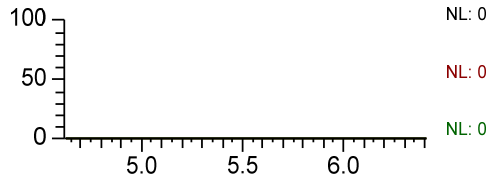

Supplementary Figure 10: Exemplary chromatograms of M4 in the measured samples.

M5

**target peak**	*m/z* 167.0371->167.0371
**conf peak 1**	*m/z* 167.0371->131.0604
**conf peak 2**	*m/z* 167.0371

doping control urine hydrolysis+LLE

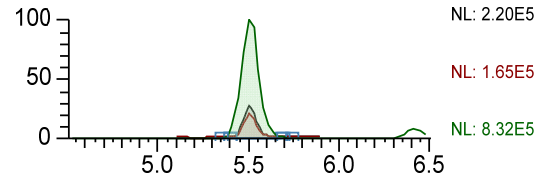

EC flow-through cell 2000 mV

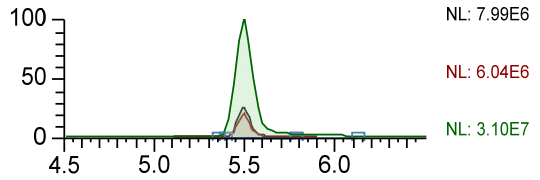

doping control urine 'dilute and shoot'

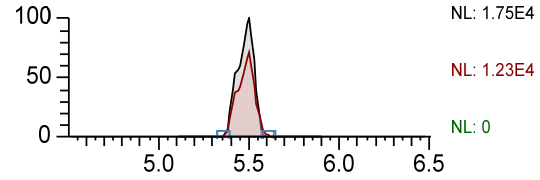

EC thin-layer cell 2100 mV

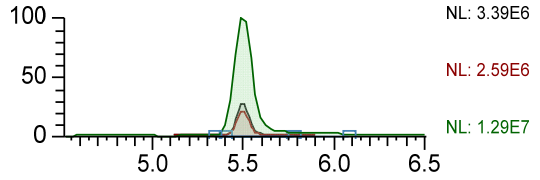

subcellular liver fractions phase I

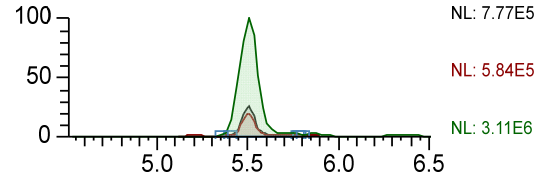

EC unoxidized

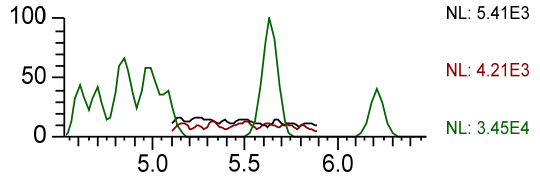

subcellular liver fractions phase I w/o fractions

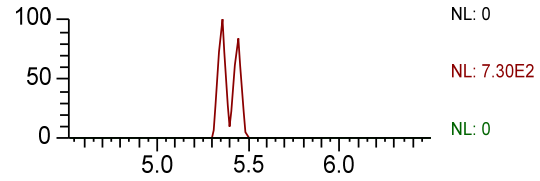

organ-on-a-chip $1 \cdot 10^6$ cells d14

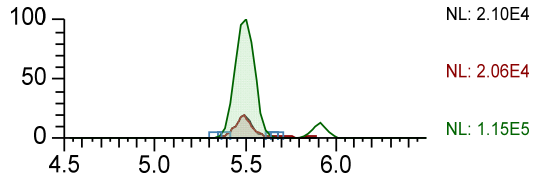

subcellular liver fractions phase I+II

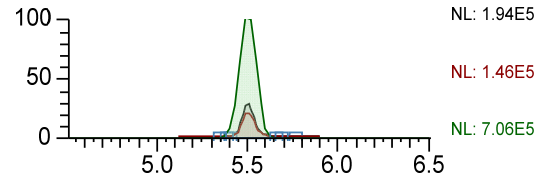

organ-on-a-chip w/o cells d14

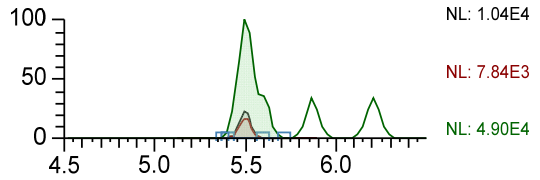

subcellular liver fractions phase I+II w/o fractions

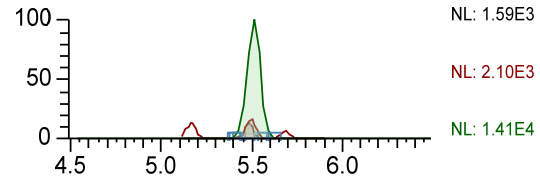

RAD140 standard

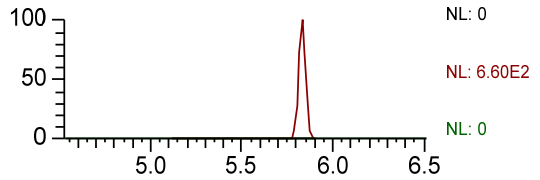

Supplementary Figure 11: Exemplary chromatograms of M5 in the measured samples.

M6a

**target peak**	*m/z* 364.0607->170.0360
**conf peak 1**	*m/z* 364.0607->193.0174
**conf peak 2**	*m/z* 364.0607->145.0407

doping control urine hydrolysis+LLE

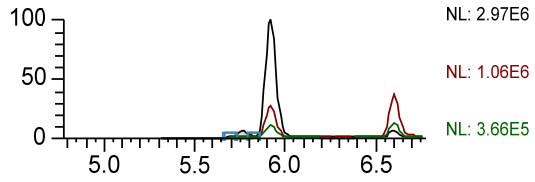

EC flow-through cell 2000 mV

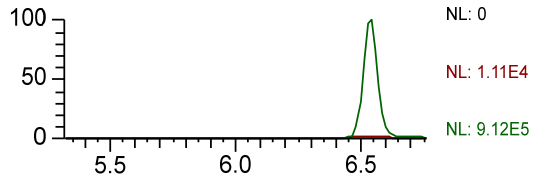

doping control urine 'dilute and shoot'

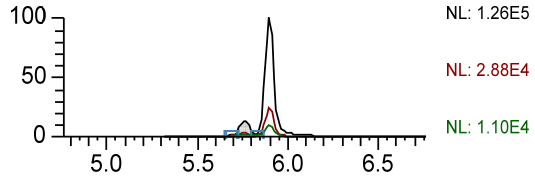

EC thin-layer cell 2100 mV

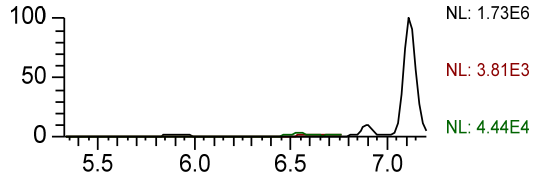

subcellular liver fractions phase I

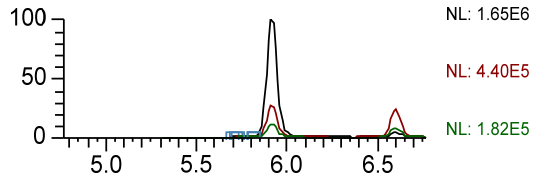

EC unoxidized

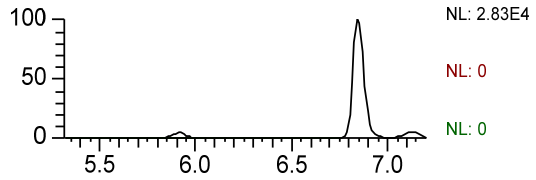

subcellular liver fractions phase I w/o fractions

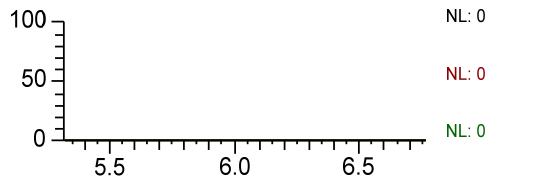

organ-on-a-chip $1 \cdot 10^6$ cells d14

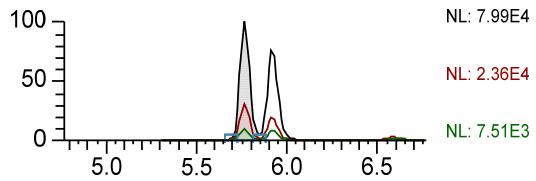

subcellular liver fractions phase I+II

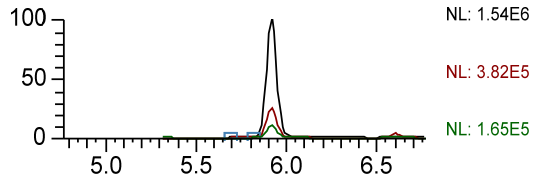

organ-on-a-chip w/o cells d14

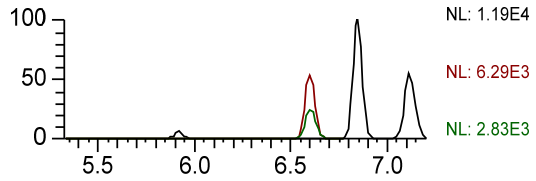

subcellular liver fractions phase I+II w/o fractions

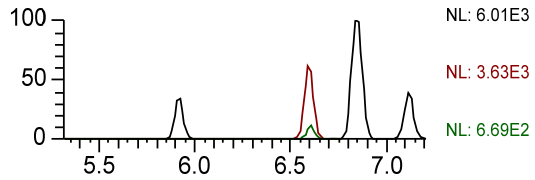

RAD140 standard

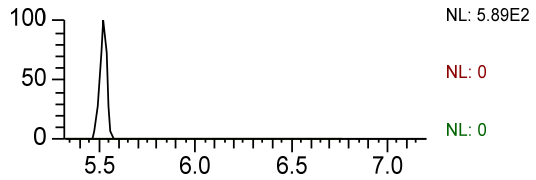

Supplementary Figure 12: Exemplary chromatograms of M6a in the measured samples.

M6b

**target peak**	*m/z* 364.0607->170.0360
**conf peak 1**	*m/z* 364.0607->193.0174
**conf peak 2**	*m/z* 364.0607->145.0407

doping control urine hydrolysis+LLE

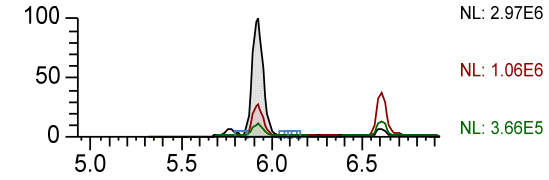

EC flow-through cell 2000 mV

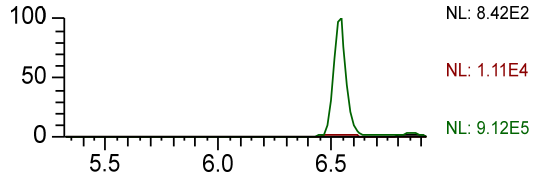

doping control urine ‘dilute and shoot’

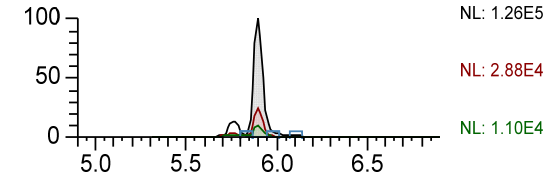

EC thin-layer cell 2100 mV

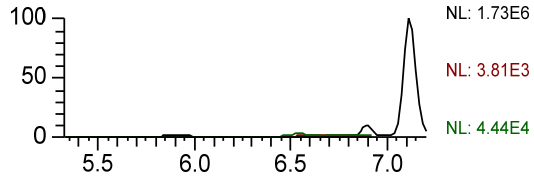

subcellular liver fractions phase I

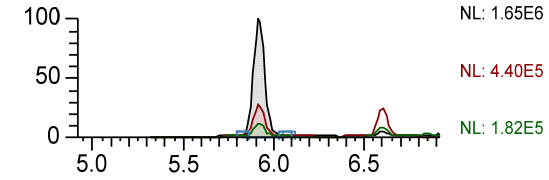

EC unoxidized

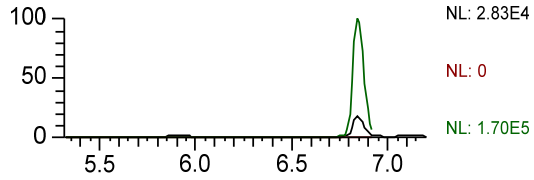

subcellular liver fractions phase I w/o fractions

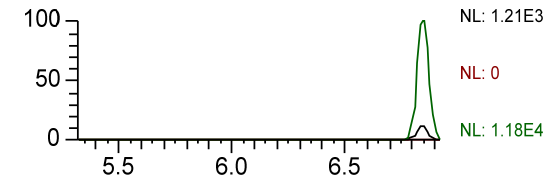

organ-on-a-chip $1 \cdot 10^6$ cells d14

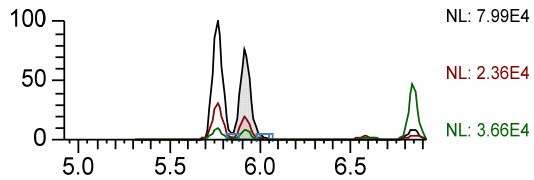

subcellular liver fractions phase I+II

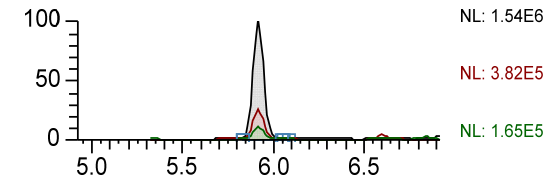

organ-on-a-chip w/o cells d14

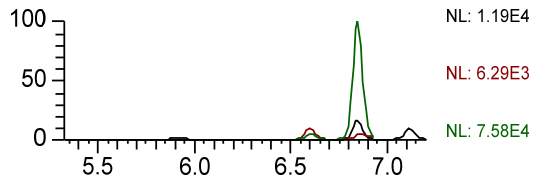

subcellular liver fractions phase I+II w/o fractions

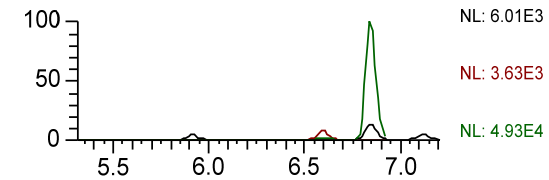

RAD140 standard

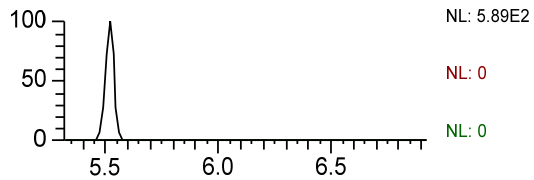

Supplementary Figure 13: Exemplary chromatograms of M6b in the measured samples.

M6c

**target peak**	*m/z* 364.0607->180.0096
**conf peak 1**	*m/z* 364.0607->193.0174
**conf peak 2**	*m/z* 364.0607->145.0407

doping control urine hydrolysis+LLE

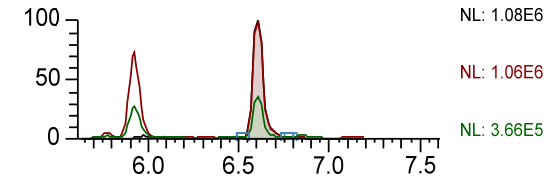

EC flow-through cell 2000 mV

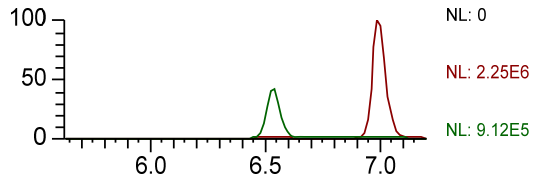

doping control urine 'dilute and shoot'

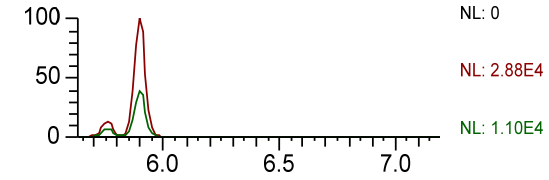

EC thin-layer cell 2100 mV

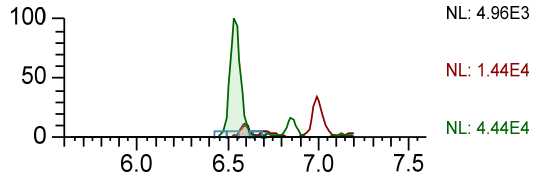

subcellular liver fractions phase I

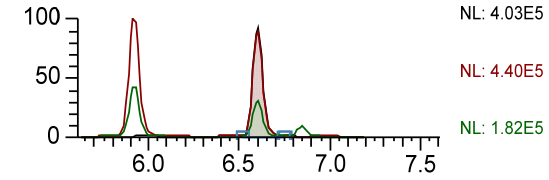

EC unoxidized

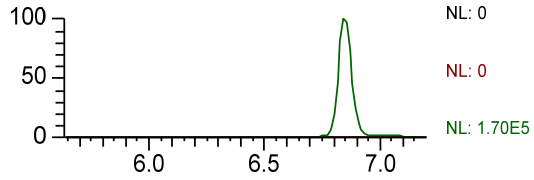

subcellular liver fractions phase I w/o fractions

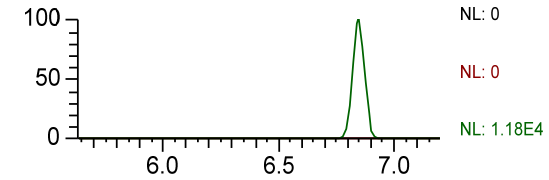

organ-on-a-chip $1 \cdot 10^6$ cells d14

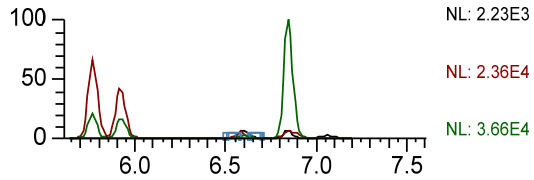

subcellular liver fractions phase I+II

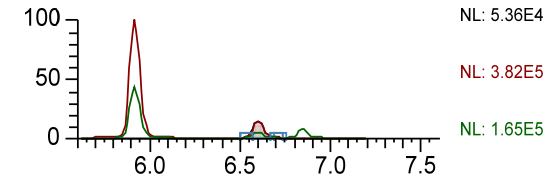

organ-on-a-chip w/o cells d14

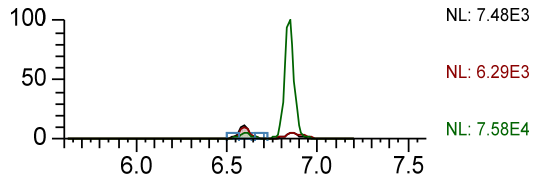

subcellular liver fractions phase I+II w/o fractions

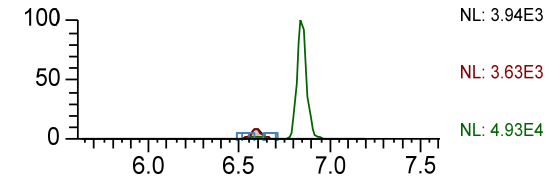

RAD140 standard

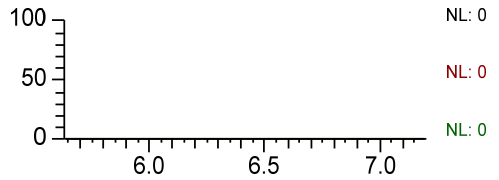

Supplementary Figure 14: Exemplary chromatograms of M6c in the measured samples.

M7

**target peak**	*m/z* 472.0488
**conf peak 1**	*m/z* 472.0488->348.0658
**conf peak 2**	*m/z* 472.0488->96.9601

doping control urine hydrolysis+LLE

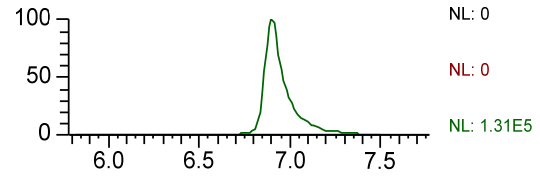

EC flow-through cell 2000 mV

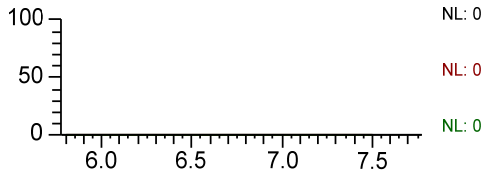

doping control urine ‘dilute and shoot’

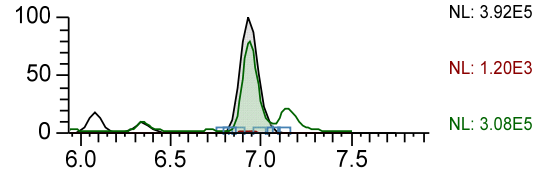

EC thin-layer cell 2100 mV

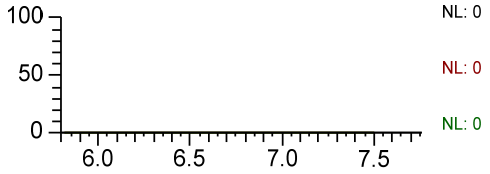

subcellular liver fractions phase I

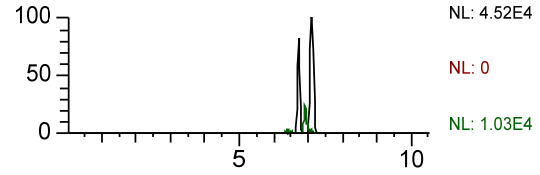

EC unoxidized

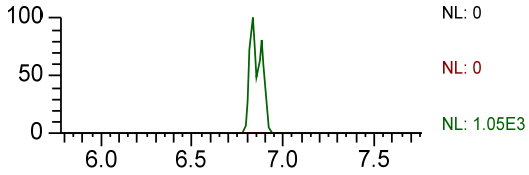

subcellular liver fractions phase I w/o fractions

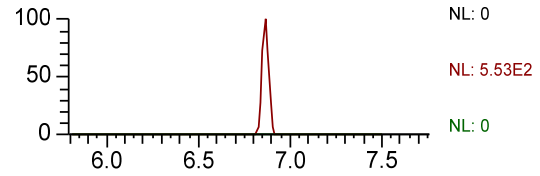

organ-on-a-chip $1 \cdot 10^6$ cells d14

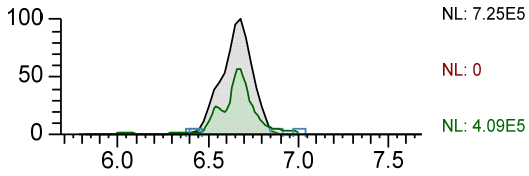

subcellular liver fractions phase I+II

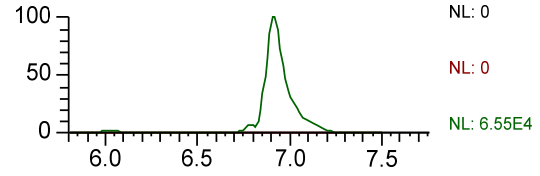

organ-on-a-chip w/o cells d14

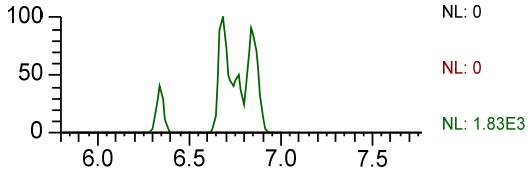

subcellular liver fractions phase I+II w/o fractions

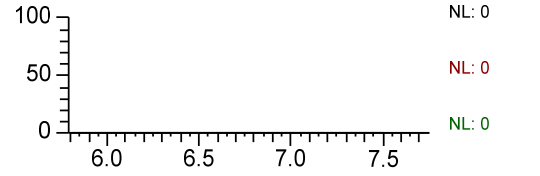

RAD140 standard

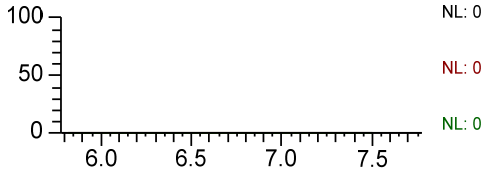

Supplementary Figure 15: Exemplary chromatograms of M7 in the measured samples.

M8

**target peak**	*m/z* 357.0495->181.0174
**conf peak 1**	*m/z* 357.0495
**conf peak 2**	*m/z* 357.0495->113.0244

doping control urine hydrolysis+LLE

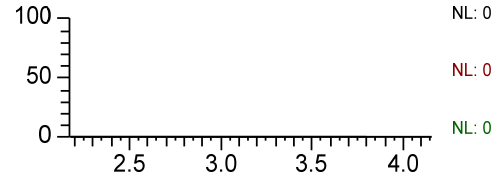

EC flow-through cell 2000 mV

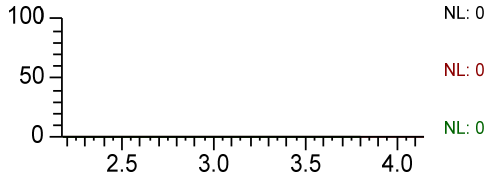

doping control urine 'dilute and shoot'

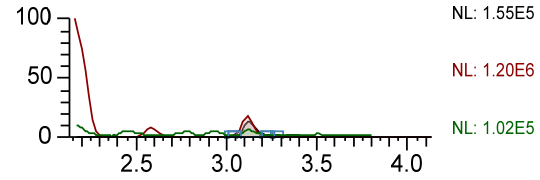

EC thin-layer cell 2100 mV

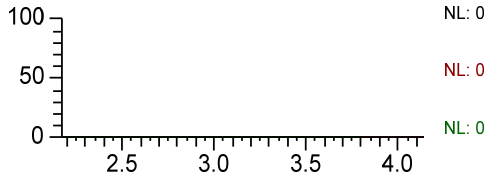

subcellular liver fractions phase I

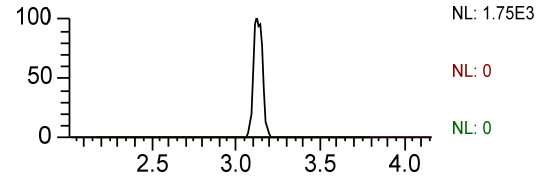

EC unoxidized

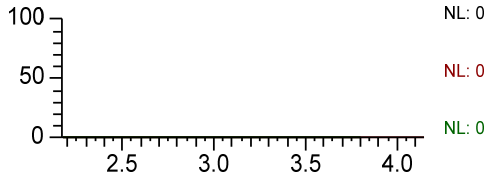

subcellular liver fractions phase I w/o fractions

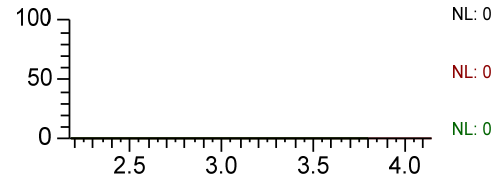

organ-on-a-chip $1 \cdot 10^6$ cells d14

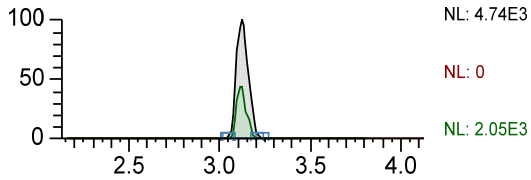

subcellular liver fractions phase I+II

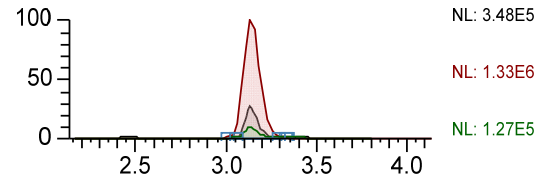

organ-on-a-chip w/o cells d14

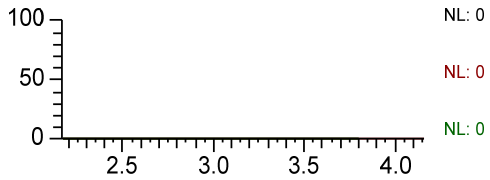

subcellular liver fractions phase I+II w/o fractions

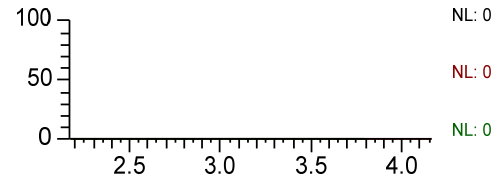

RAD140 standard

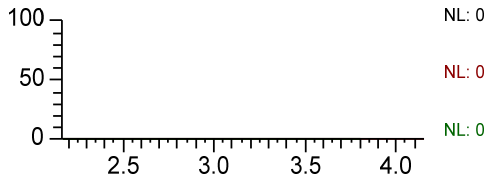

Supplementary Figure 16: Exemplary chromatograms of M8 in the measured samples.

M9

**target peak**	*m/z* 392.0906
**conf peak 1**	*m/z* 392.0906->177.0214
**conf peak 2**	*m/z* 392.0906->142.0526

doping control urine hydrolysis+LLE

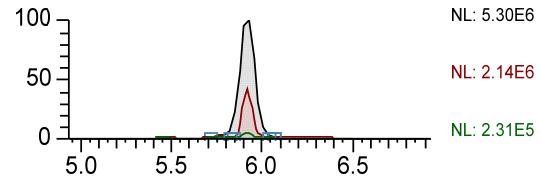

EC flow-through cell 2000 mV

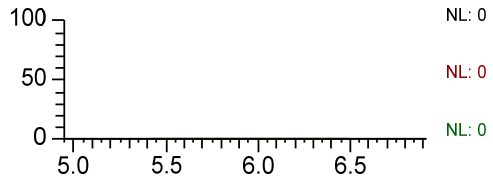

doping control urine 'dilute and shoot'

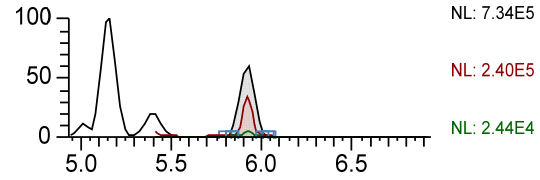

EC thin-layer cell 2100 mV

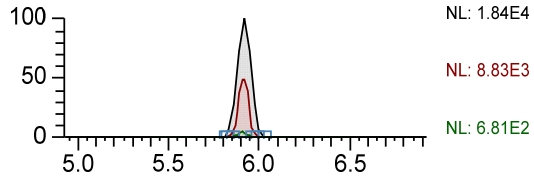

subcellular liver fractions phase I

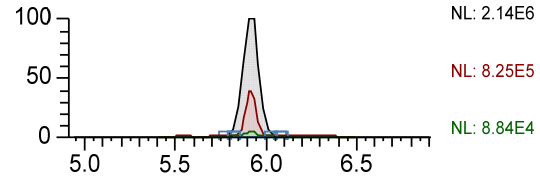

EC unoxidized

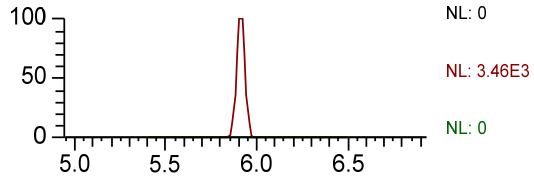

subcellular liver fractions phase I w/o fractions

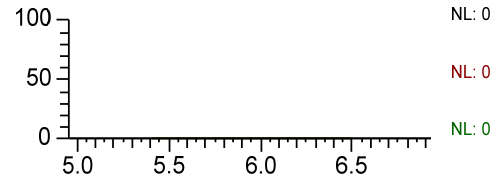

organ-on-a-chip $1 \cdot 10^6$ cells d14

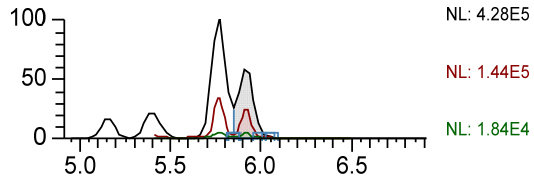

subcellular liver fractions phase I+II

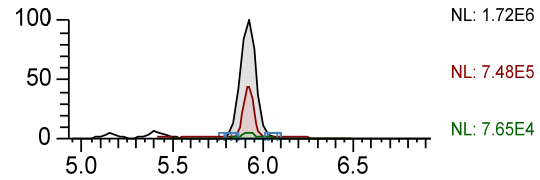

organ-on-a-chip w/o cells d14

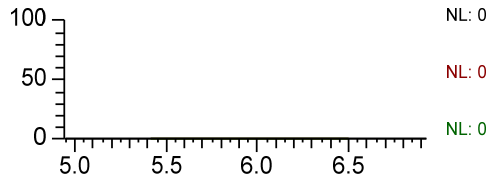

subcellular liver fractions phase I+II w/o fractions

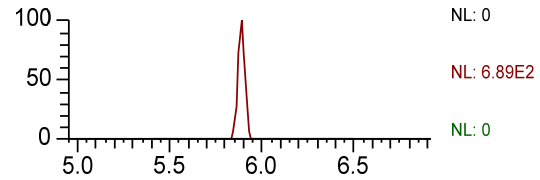

RAD140 standard

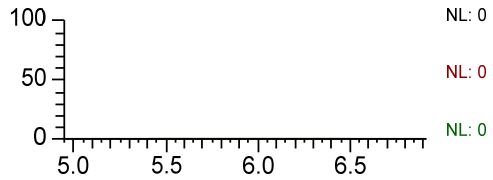

Supplementary Figure 17: Exemplary chromatograms of M9 in the measured samples.

M10a

**target peak**	*m/z* 362.0450->145.0407
**conf peak 1**	*m/z* 362.0450->362.0450
**conf peak 2**	*m/z* 362.0450->204.0101

doping control urine hydrolysis+LLE

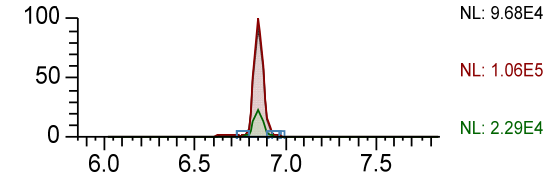

doping control urine 'dilute and shoot'

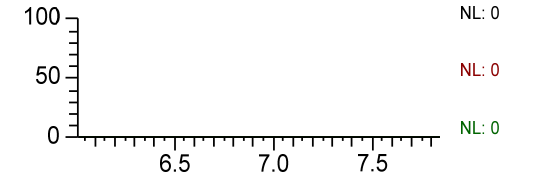

subcellular liver fractions phase I

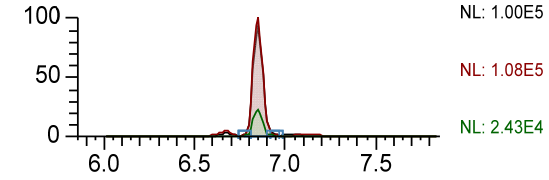

subcellular liver fractions phase I w/o fractions

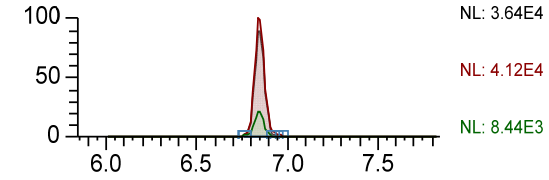

subcellular liver fractions phase I+II

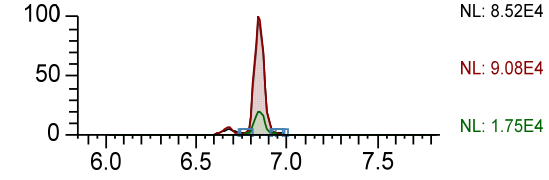

subcellular liver fractions phase I+II w/o fractions

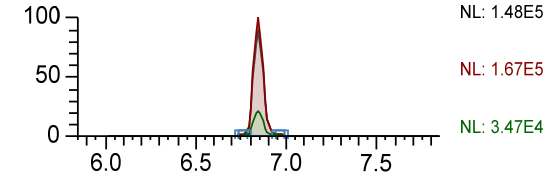

EC flow-through cell 2000 mV

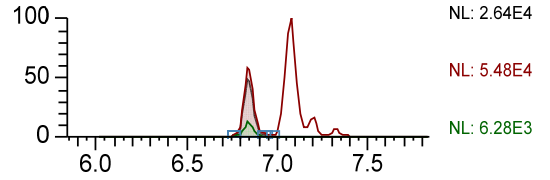

EC thin-layer cell 2100 mV

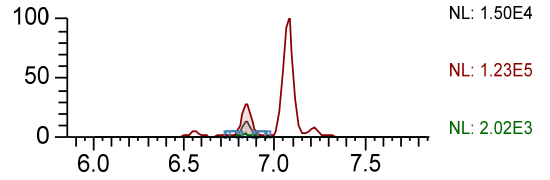

EC unoxidized

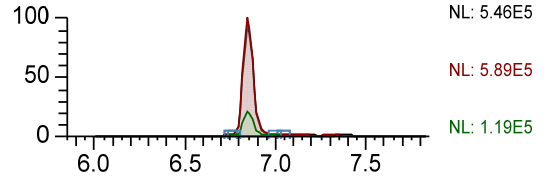

organ-on-a-chip $1 \cdot 10^6$ cells d14

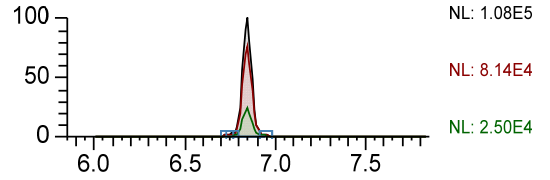

organ-on-a-chip w/o cells d14

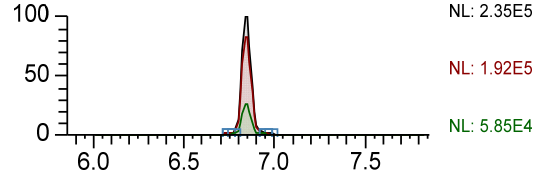

RAD140 standard

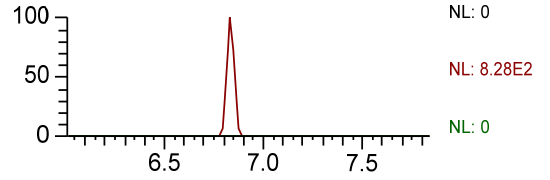

Supplementary Figure 18: Exemplary chromatograms of M10a in the measured samples.

M10b

**target peak**	*m/z* 362.0450
**conf peak 1**	*m/z* 362.0450->170.0360
**conf peak 2**	N/A

doping control urine hydrolysis+LLE

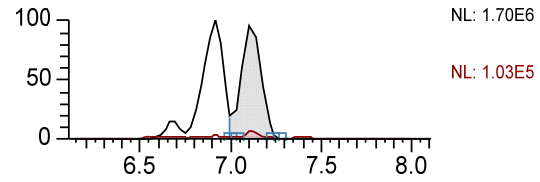

EC flow-through cell 2000 mV

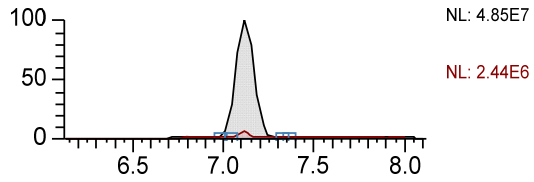

doping control urine ‘dilute and shoot’

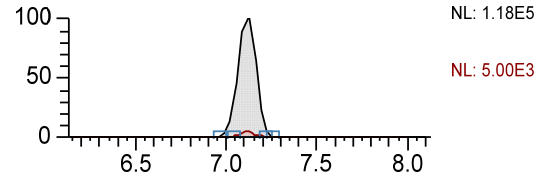

EC thin-layer cell 2100 mV

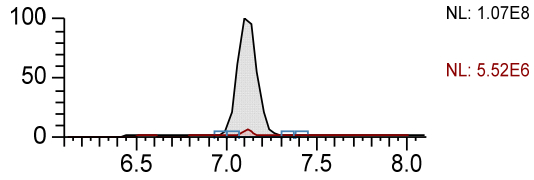

subcellular liver fractions phase I

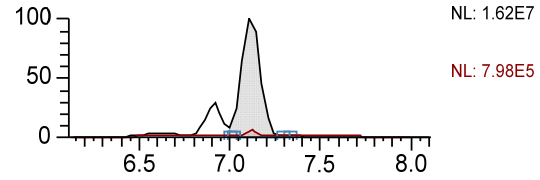

EC unoxidized

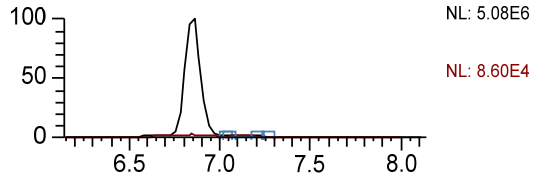

subcellular liver fractions phase I w/o fractions

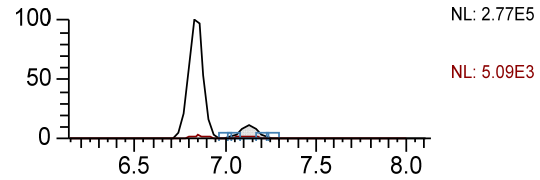

organ-on-a-chip $1 \cdot 10^6$ cells d14

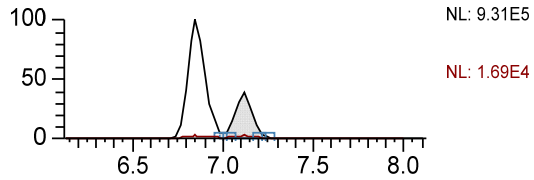

subcellular liver fractions phase I+II

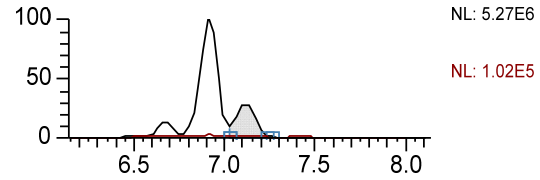

organ-on-a-chip w/o cells d14

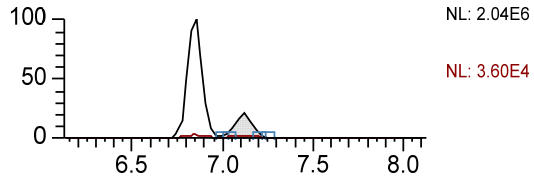

subcellular liver fractions phase I+II w/o fractions

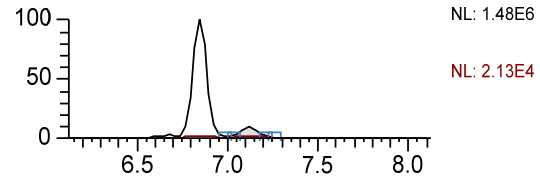

RAD140 standard

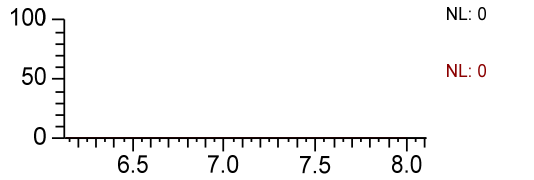

Supplementary Figure 19: Exemplary chromatograms of M10b in the measured samples.

M11

**target peak**	*m/z* 374.0814->170.0360
**conf peak 1**	*m/z* 374.0814->127.0302
**conf peak 2**	*m/z* 374.0814->374.0814

doping control urine hydrolysis+LLE

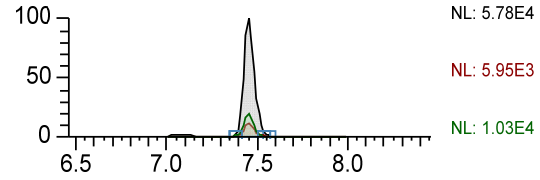

EC flow-through cell 2000 mV

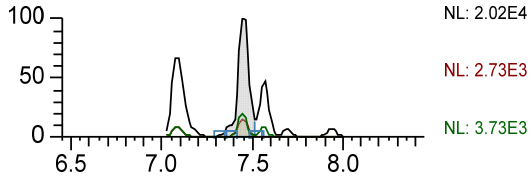

doping control urine 'dilute and shoot'

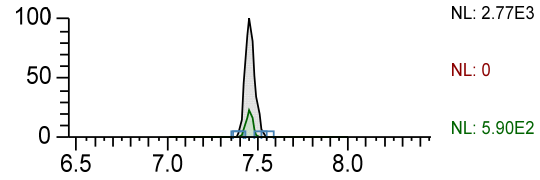

EC thin-layer cell 2100 mV

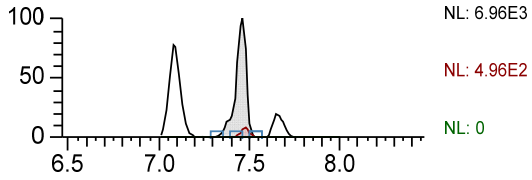

subcellular liver fractions phase I

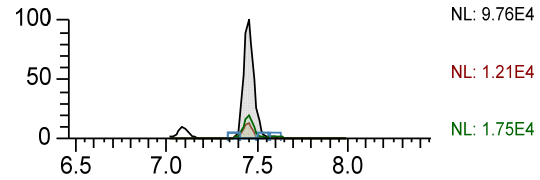

EC unoxidized

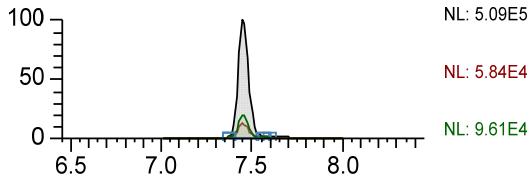

subcellular liver fractions phase I w/o fractions

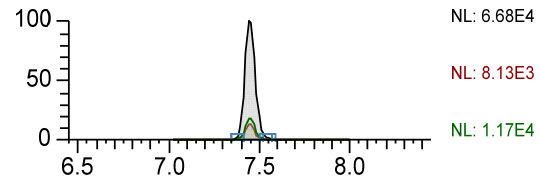

organ-on-a-chip $1 \cdot 10^6$ cells d14

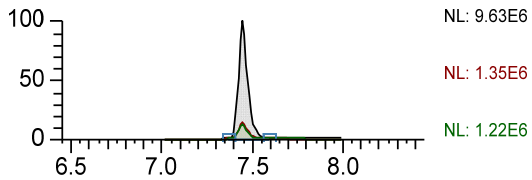

subcellular liver fractions phase I+II

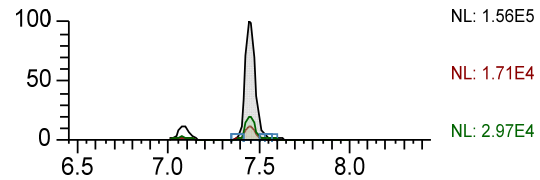

organ-on-a-chip w/o cells d14

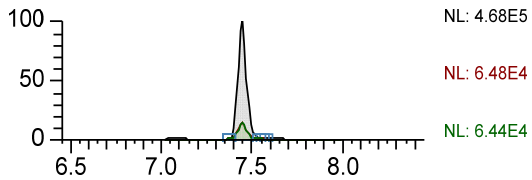

subcellular liver fractions phase I+II w/o fractions

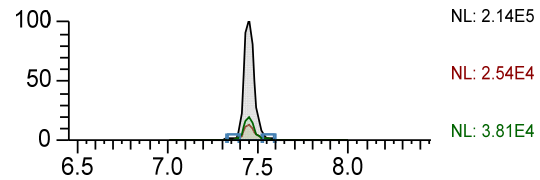

RAD140 standard

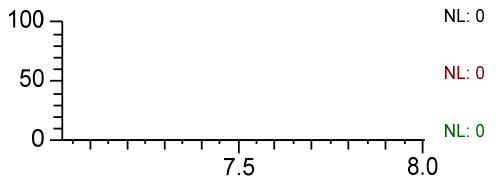

Supplementary Figure 20: Exemplary chromatograms of M11 in the measured samples.

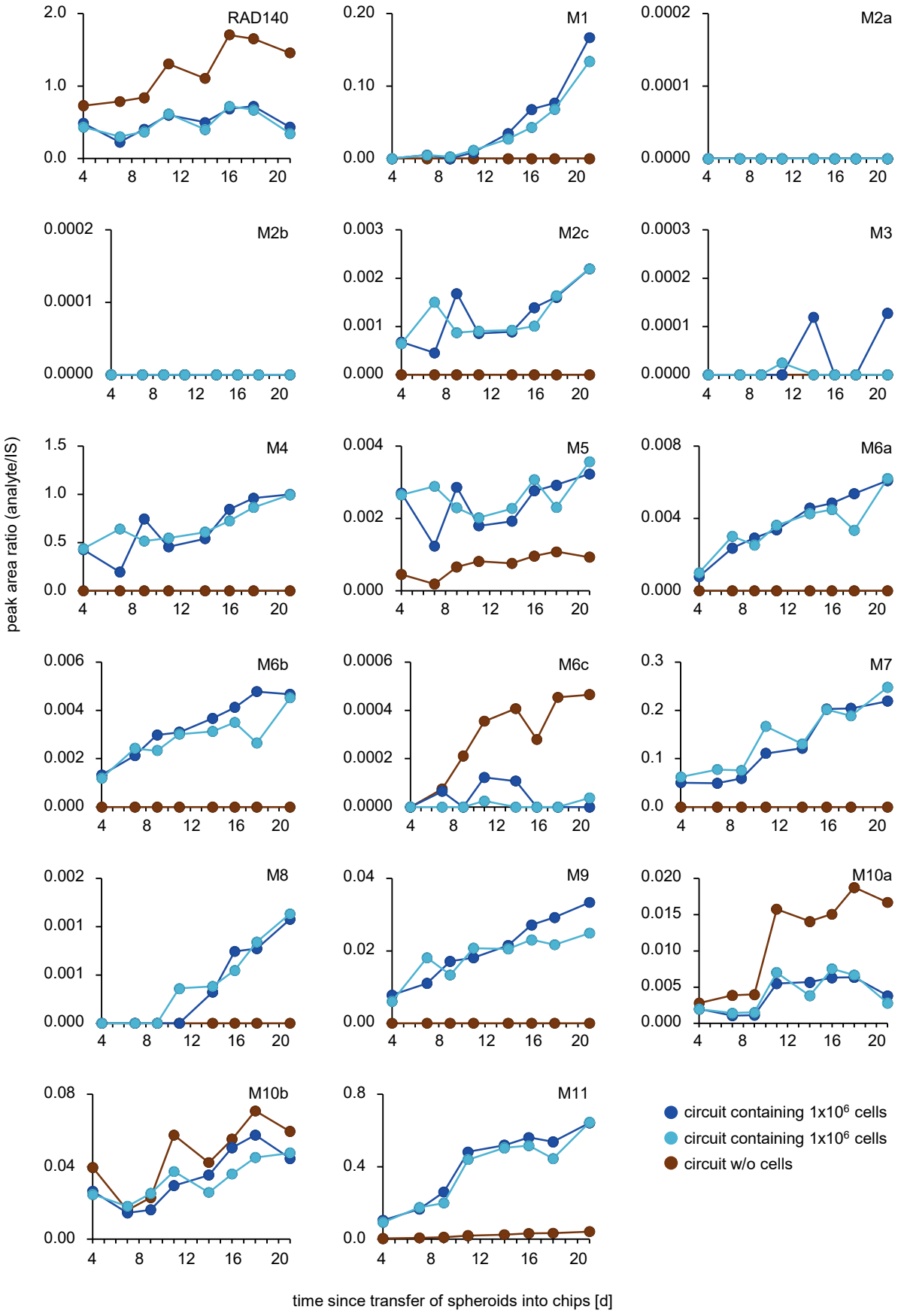

Supplementary Figure 21: Graphs depicting the intensity of all detected metabolites as analyte/IS peak area ratio over the course of the organ-on-a-chip experiment.

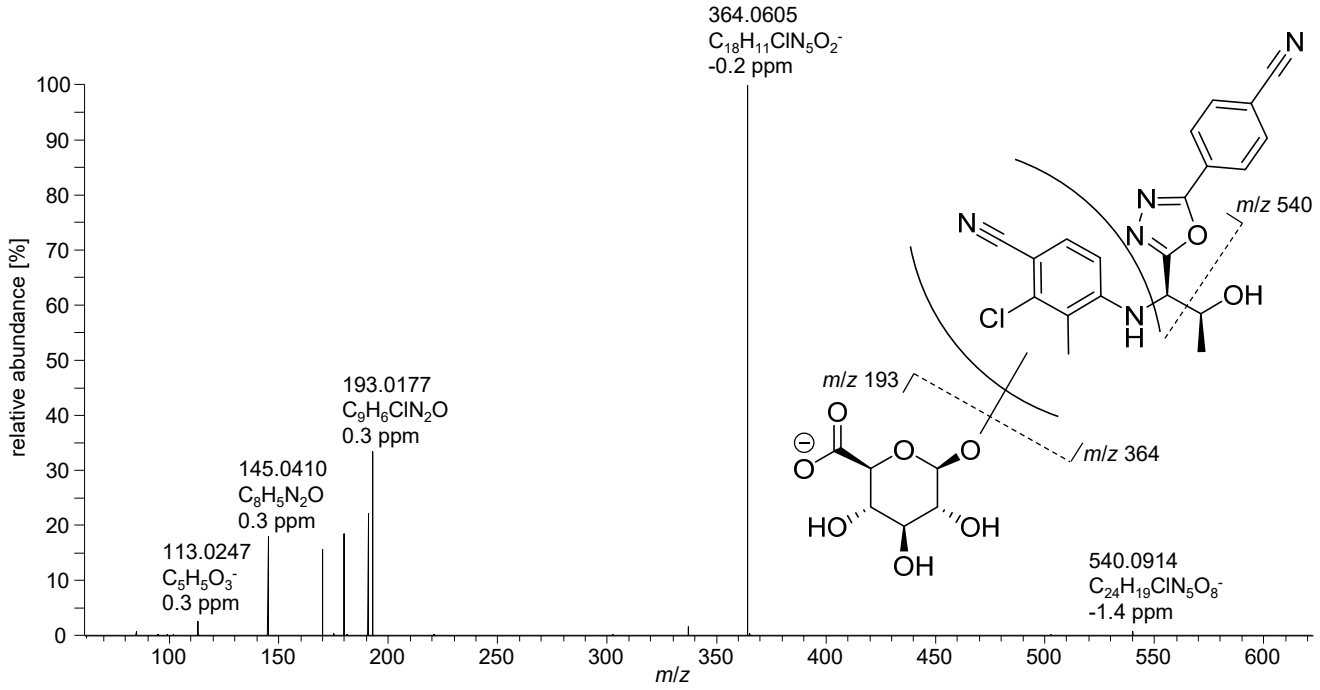

Supplementary Figure 22: HRMS/MS spectrum and proposed structure of M2c, measured in a sample after phase I and II incubation with subcellular liver fractions. Prominent ion transitions are marked with the measured *m*/z, the proposed molecular formula and the deviation of measured m/z to monoisotopic mass. The precursor *m*/z 584.1190 was isolated with a window of 1 *m*/z and dissociated with NCE 30%.

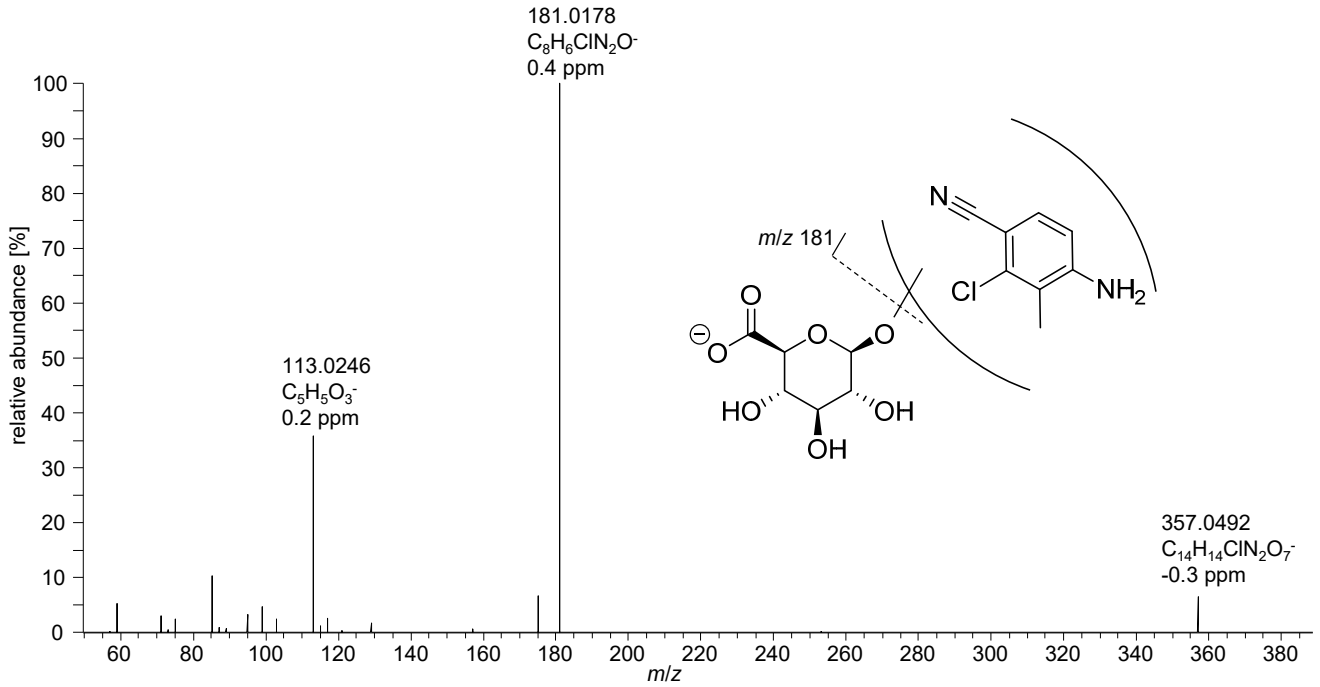

Supplementary Figure 23: HRMS/MS spectrum and proposed structure of M8, measured in a sample after phase I and II incubation with subcellular liver fractions. Prominent ion transitions are marked with the measured *m*/z, the proposed molecular formula and the deviation of measured m/z to monoisotopic mass. The precursor *m*/z 357.0495 was isolated with a window of 1 *m*/z and dissociated with NCE 30%.

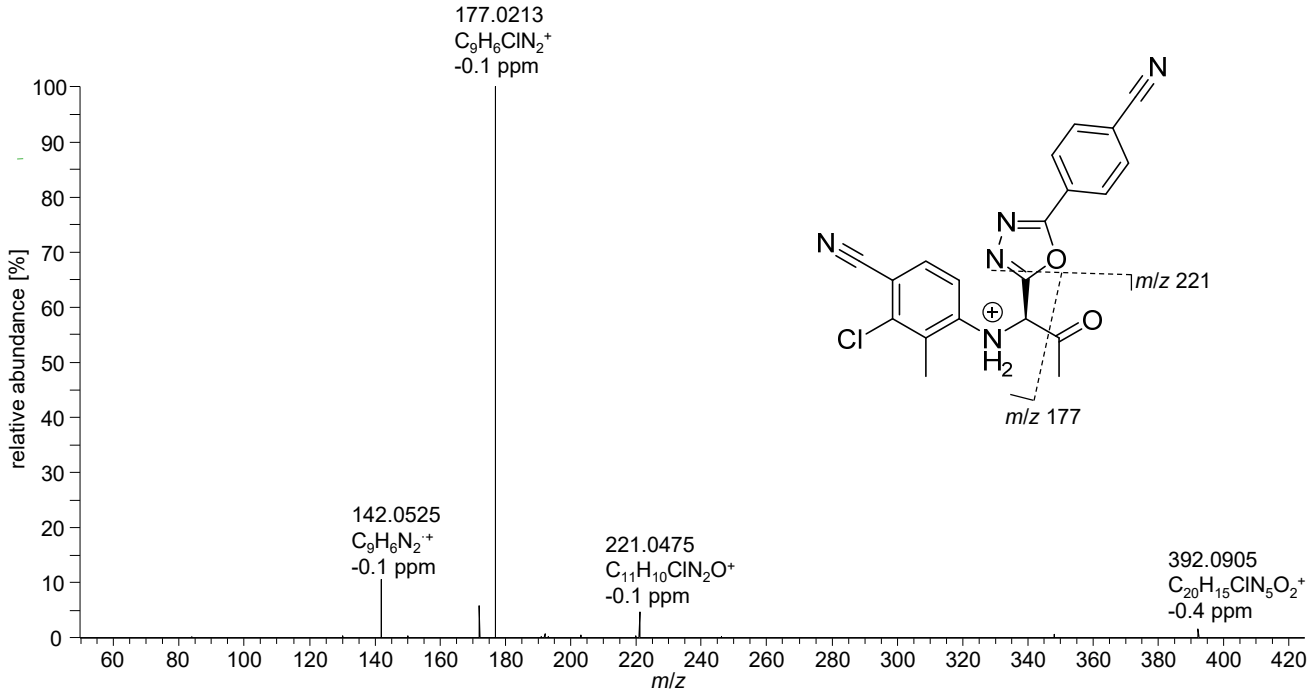

Supplementary Figure 24: HRMS/MS spectrum and proposed structure of M9, measured in a routine doping control urine sample after sample preparation with hydrolysis and LLE. Prominent ion transitions are marked with the measured *m/z*, the proposed molecular formula and the deviation of measured *m/z* to monoisotopic mass. The precursor *m/z* 392.0909 was isolated with a window of 1 *m/z* and dissociated with NCE 30%.

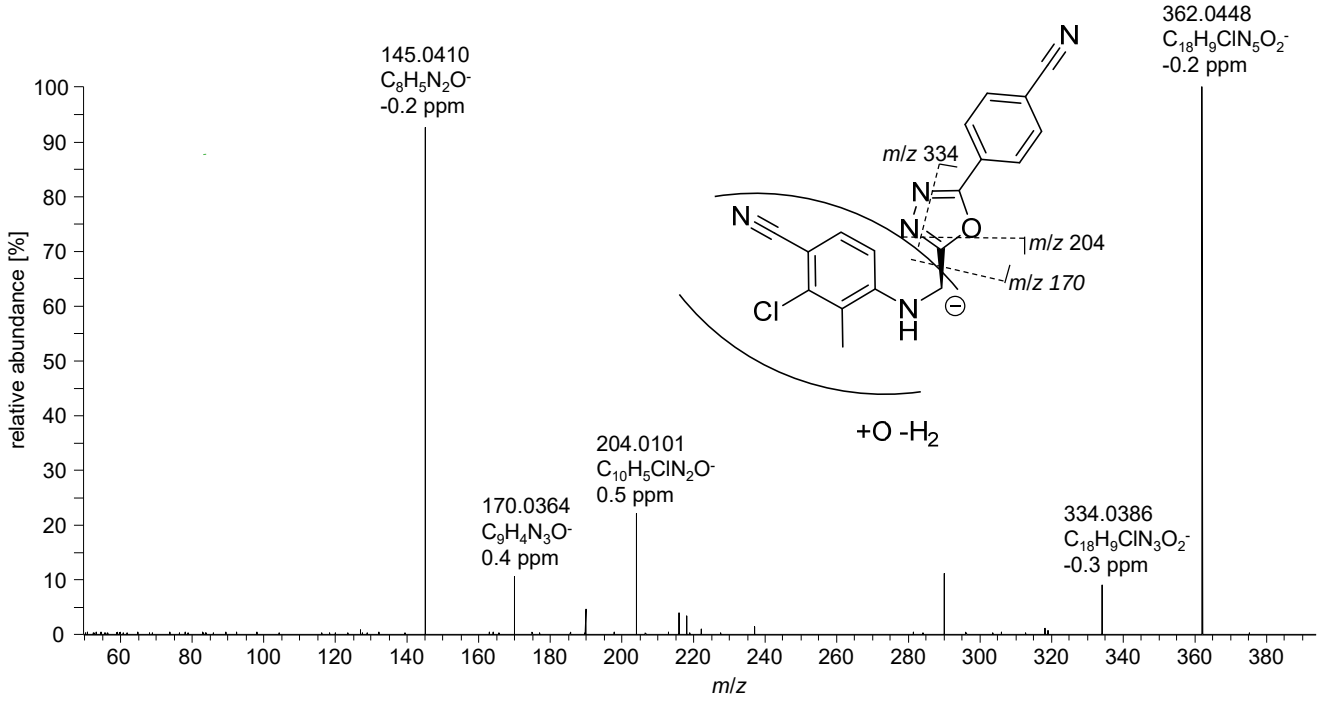

Supplementary Figure 25: HRMS/MS spectrum and proposed structure of M10a, measured in a routine doping control urine sample after sample preparation with hydrolysis and LLE. Prominent ion transitions are marked with the measured *m/z*, the proposed molecular formula and the deviation of measured *m/z* to monoisotopic mass. The precursor *m/z* 362.0450 was isolated with a window of 1 *m/z* and dissociated with NCE 30%.

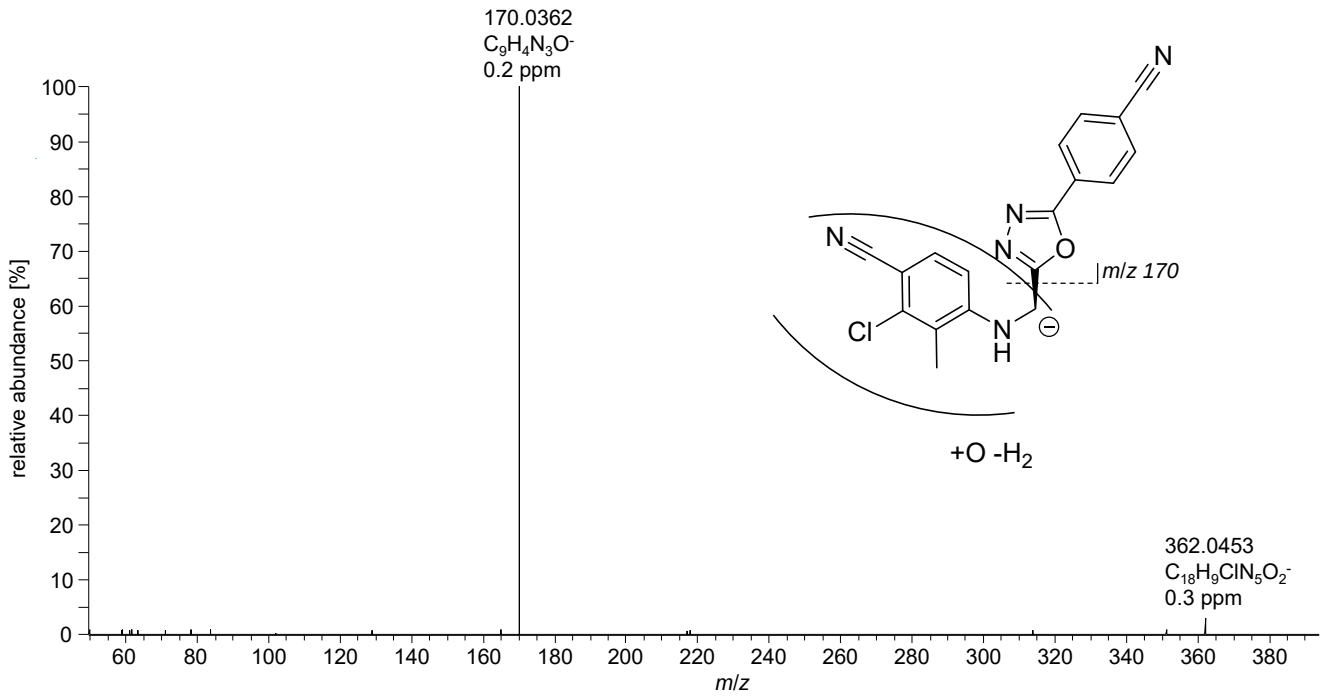

Supplementary Figure 26: HRMS/MS spectrum and proposed structure of M10b, measured in sample after oxidation at 2500 mV in a thin-layer EC cell. Prominent ion transitions are marked with the measured *m*/*z*, the proposed molecular formula and the deviation of measured *m*/*z* to monoisotopic mass. The precursor *m*/*z* 362.0450 was isolated with a window of 1 *m*/*z* and dissociated with NCE 30%.

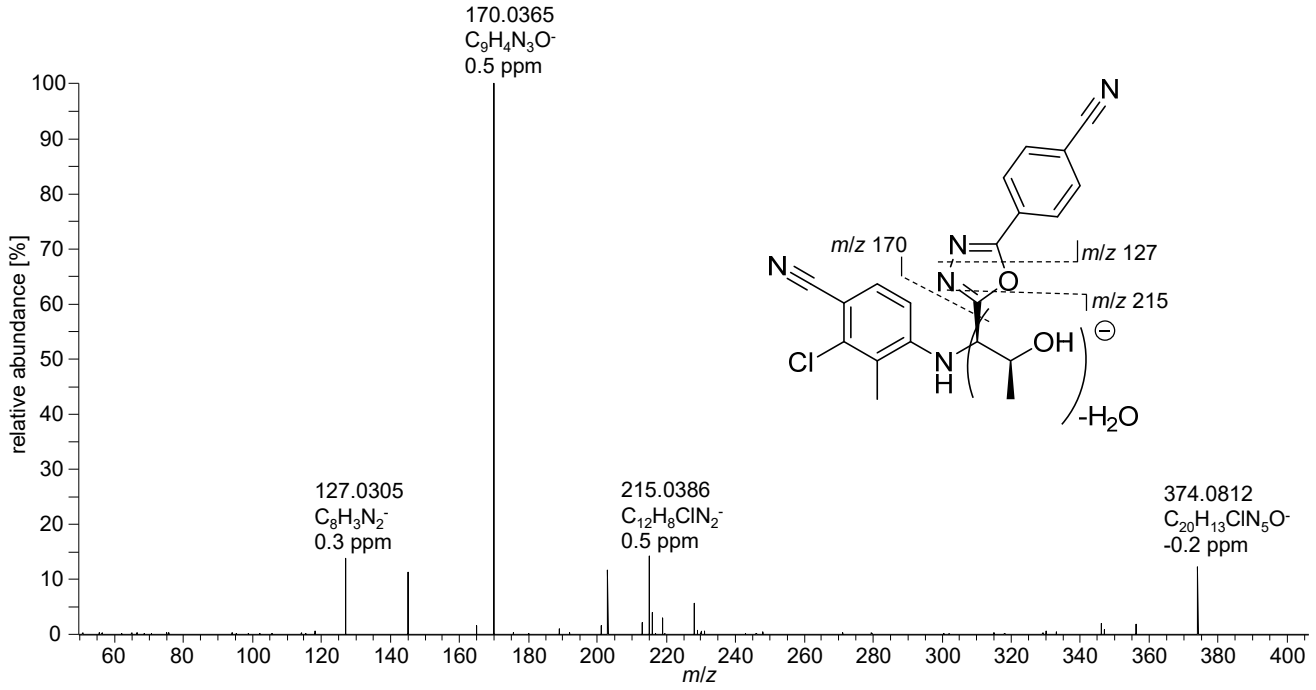

Supplementary Figure 27: HRMS/MS spectrum and proposed structure of M11, measured in a sample after incubation with liver spheroids (1x10^6 cells) in the organ-on-a-chip platform on day 14 of culture. Prominent ion transitions are marked with the measured *m*/*z*, the proposed molecular formula and the deviation of measured *m*/*z* to monoisotopic mass. The precursor *m*/*z* 362.0450 was isolated with a window of 1 *m*/*z* and dissociated with NCE 30%.

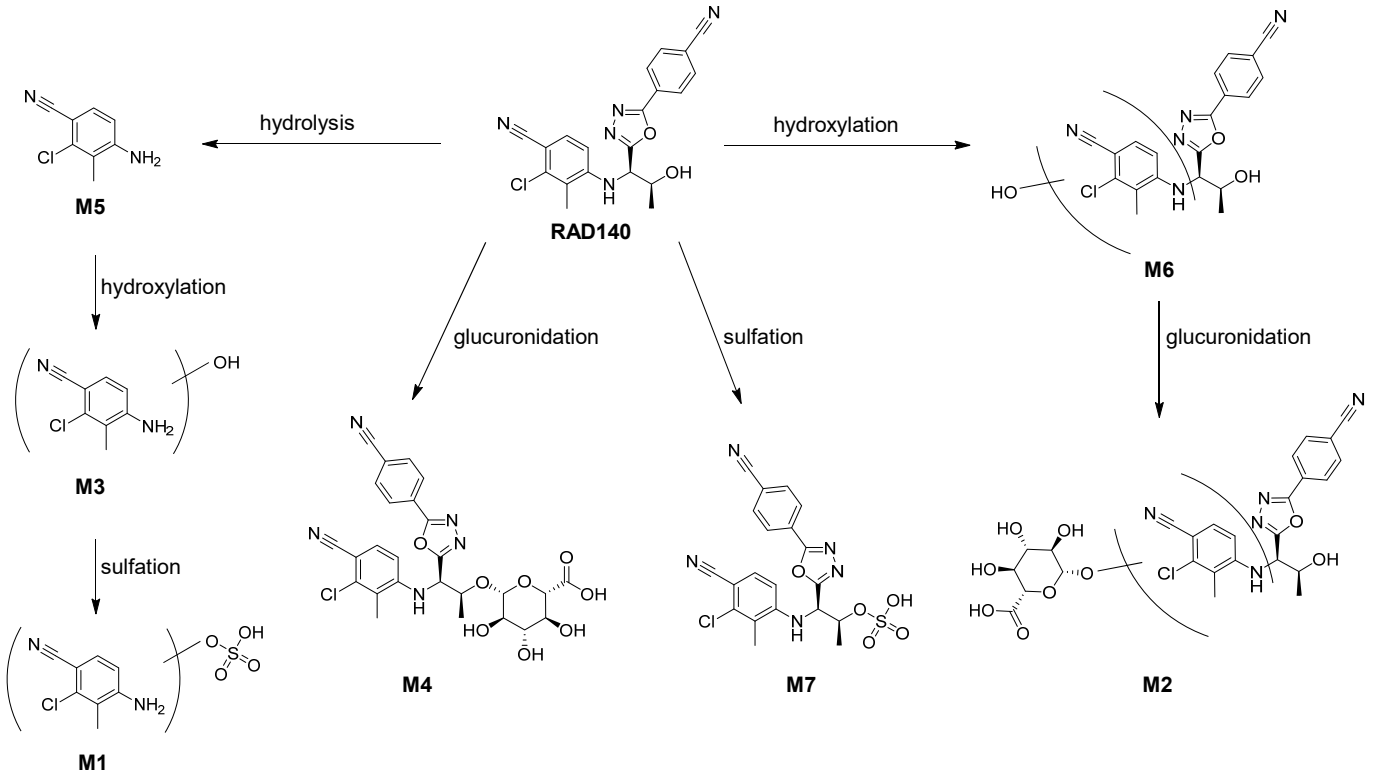

Supplementary Figure 28: Structures and metabolic pathways of the previously described metabolites M1, M2, M3, M4, M5, M6 and M7 (adapted from [1]).

References:

1. Wagener F, Euler L, Görgens C, Guddat S, Thevis M. Human In Vivo Metabolism and Elimination Behavior of Micro-Dosed Selective Androgen Receptor Modulator RAD140 for Doping Control Purposes. Metabolites. 2022;12(7):666. https://doi.org/10.3390/metabo12070666
